# PRMT7‐mediated arginine methylation of FOXK activates Wnt/β‐catenin signalling to drive doxorubicin resistance in diffuse large B‐cell lymphoma

**DOI:** 10.1002/ctm2.70802

**Published:** 2026-09-02

**Authors:** Xiaoyan Zhao, Jiasi Zhang, Yao Teng, Yaqin Wang, Wen Yu, Yan Sun, Zhou Wu, Aiguo Liu

**Affiliations:** ^1^ Department of Pediatrics, Tongji Hospital, Tongji Medical College Huazhong University of Science and Technology Wuhan China; ^2^ Department of Thoracic Surgery, Union Hospital, Tongji Medical College Huazhong University of Science and Technology Wuhan China; ^3^ Department of Kidney Disease General Hospital of Central Theater Command Wuhan China

**Keywords:** diffuse large B‐cell lymphoma, doxorubicin resistance, Forkhead box K, methylation, protein arginine methyltransferase 7

## Abstract

**Background:**

Doxorubicin, an anthracycline chemotherapeutic agent, is widely used in diffuse large B‑cell lymphoma (DLBCL) treatment, yet its clinical efficacy is often compromised by drug resistance. Protein arginine methyltransferase 7 (PRMT7) is a methyltransferase implicated in tumourigenesis and cancer progression. However, its precise role and underlying mechanisms in DLBCL progression and doxorubicin resistance remain unclear.

**Methods:**

We analysed PRMT7 expression in DLBCL cell lines and patient specimens using bioinformatic databases, western blotting, reverse Transcription Polymerase Chain Reactionand immunohistochemistry. Functional studies were performed in DLBCL cell lines through CRISPR/Cas9‑mediated knockout and overexpression systems, combined with in vitro assays for proliferation, apoptosis and doxorubicin sensitivity, as well as in vivo xenograft models. Mechanistically, co‑immunoprecipitation, arginine methylation assays and immunofluorescence were employed to characterise the PRMT7–Forkhead box K (FOXK)1/2–Dishevelled Segment Polarity Protein 2 (DVL2) axis and its modulation of Wnt/ (beta) β‑catenin signalling. In addition, we designed and evaluated FOXK‑methylation‑competitive inhibitory peptides for their capacity to sensitise DLBCL cells to doxorubicin.

**Results:**

Our study revealed that PRMT7 is upregulated in DLBCL and promotes both tumour proliferation and doxorubicin resistance. Conversely, knockdown of PRMT7 inhibited DLBCL cell proliferation and enhanced sensitivity to doxorubicin. Mechanistically, PRMT7 catalyses arginine methylation of FOXK1 at arginine (R) 191 and FOXK2 at R144, which markedly increases their binding affinity for DVL2 and facilitates DVL2 nuclear translocation. This event leads to constitutive activation of the Wnt/β‑catenin signalling pathway. Importantly, we developed a FOXK‑derived peptide that competitively inhibits FOXK methylation, suppresses Wnt/β‑catenin signalling and significantly potentiates the antitumour efficacy of doxorubicin in DLBCL.

**Conclusion:**

Our findings indicate that the PRMT7/FOXK/DVL2/Wnt‐β‐catenin axis serves as a novel driver of DLBCL progression and doxorubicin resistance. Targeting the PRMT7–FOXK methylation interface may offer a potential therapeutic approach for overcoming doxorubicin resistance in DLBCL, although further validation in clinical settings is warranted.

**Key points:**

- Upregulated PRMT7 drives DLBCL progression and doxorubicin resistance
- PRMT7 methylates FOXK1 at arginine 191 and FOXK2 at arginine 144
- FOXK1/2 methylation enhances interaction with DVL2 and promotes nuclear translocation
- FOXK1/2 methylation dactivate Wnt/ß-catenin signaling through DVL2 nuclear entry
- A novel peptide inhibitory blocks FOXK1/2 methylation and restores doxorubicin sensitivity

## Graphical abstract

The high expression of PRMT7 enhances its interaction with DVL2 by methylating arginine residues on FOXK1/FOXK2. This process promotes the nuclear translocation of DVL2, thereby aberrantly activating the Wnt/β‐catenin signalling pathway, which ultimately drives the development of doxorubicin resistance in DLBCL. Importantly, FOXK methylation‐competitive inhibitory peptides effectively reverses this resistance.

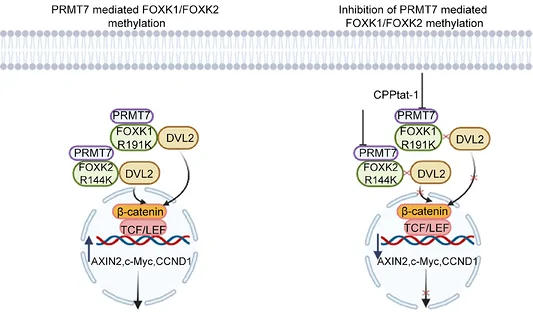

## 1 INTRODUCTION

Diffuse large B‐cell lymphoma (DLBCL) is the most common subtype of non‐Hodgkin lymphoma.^1^, ^2^, ^3^ Although frontline chemotherapy is effective, a substantial proportion of patients develop resistance, particularly to the anthracycline agent doxorubicin (DOX).^4^ This resistance often leads to treatment failure and poor prognosis.^5^, ^6^, ^7^ However, the underlying mechanisms remain poorly understood, highlighting the urgent need to identify novel therapeutic targets to improve patient outcomes.

Protein arginine methyltransferase 7 (PRMT7) is a type III arginine methyltransferase that catalyses monomethylation of arginine residues and has garnered considerable interest in recent years.^8^, ^9^, ^10^ It participates in disease pathogenesis by modulating multiple signalling pathways.^10^, ^11^ In oncology, PRMT7 contributes to the progression of various malignancies, exemplified by its regulation of leukaemia stem cell behaviour in chronic myeloid leukaemia.^12^ Beyond cancer, PRMT7 is also involved in non‑neoplastic processes, such as the monomethylation‑mediated innate immune response to viral infection via Mitochondrial Antiviral Signaling Protein,^11^ as well as cardiac hypertrophy and fibrosis.^13^ However, its role in DLBCL, particularly its involvement in DOX resistance, remains largely unknown.

Forkhead box K 1(FOXK1) and FOXK2 are evolutionarily conserved members of the FOX transcription factor family and have been shown to regulate key biological processes, including autophagy^14^ and glycolysis.^15^ Beyond their established roles in tumourigenesis and cancer progression,^16^ recent studies have also implicated FOXK1/2 in physiological processes such as cardiomyocyte proliferation.^17^

In this study, we show that PRMT7 upregulation induces DOX resistance in DLBCL cells. Mechanistically, PRMT7 methylates FOXK1 and FOXK2 at arginine (R) residues, which enhances their respective binding to Dishevelled 2 (DVL2). These interactions facilitate DVL2 nuclear translocation, leading to sustained Wnt/(Beta) β‑catenin activation and ultimately conferring chemoresistance.

## 2 METHODS

### 2.1 Patient's samples, cell lines and cell culture

The OCI‑Ly3, SUDHL‑2, SUDHL‑6 and HEK‑293T cell lines were purchased from Procell Life Science & Technology Co., Ltd (Wuhan, China), and the OCI‑Ly1 cell line was obtained from Nanjing CObIOER Biotechnology Co., Ltd (Nanjing, China). Peripheral blood from 12 healthy donors was collected, and B cells were isolated as normal controls for determining PRMT7 expression levels in DLBCL cell lines. Archived paraffin‑embedded tissue samples were collected from 63 newly diagnosed DLBCL patients and 15 reactive hyperplastic lymph node specimens (as non‑malignant controls) between May 2022 and April 2023, to evaluate PRMT7 expression. All patients received first‑line R‑CHOP chemotherapy. Specimen collection was performed in accordance with informed consent and complied with the ethical principles of the Declaration of Helsinki. The study protocol was approved by the Ethics Committee of Union Hospital, Huazhong University of Science and Technology.

All cells were cultured at 37°C in a humidified atmosphere containing 5% CO_2_. SUDHL‑2 and SUDHL‑6 cells were maintained in RPMI‑1640 (Gibco, USA) supplemented with 10% foetal bovine serum (FBS) and 1% penicillin–streptomycin. OCI‑Ly1 cells were cultured in IMDM (Gibco) containing 20% FBS and 1% penicillin–streptomycin. OCI‑Ly3 cells were grown in RPMI‑1640 with 20% FBS and 1% penicillin–streptomycin. HEK‑293T cells were cultured in DMEM supplemented with 10% FBS and 1% penicillin–streptomycin. All cell lines were authenticated by short tandem repeat profiling at Procell Life Science & Technology Co., Ltd. Plasmocin‑Mycoplasma Elimination Reagent (#ant‑mpp; InvivoGen) was routinely added to the culture medium to prevent mycoplasma contamination. Mycoplasma status was monitored every 3 months using the PlasmoTest‑Mycoplasma Detection Kit (#rep‑pt1; InvivoGen, distributed by MingRui Biotech Co., Ltd), and all tests remained negative throughout the study period.

### 2.2 Establishment of DOX‐resistant DLBCL cell lines

SUDHL2 and SUDHL6 cells in the logarithmic growth phase were used to establish DOX‐resistant cell lines via a stepwise dose‐escalation method. Based on preliminary half‑maximal inhibitory concentration (IC50) values of the parental cells, the initial induction concentrations were set at 15 nM for SUDHL2 and 12 nM for SUDHL6. The procedure was as follows: cells were cultured in complete medium containing the corresponding concentration of DOX. Once they resumed normal proliferation and grew stably, they were transferred to drug‐free medium and passaged twice to recover viability, after which the DOX concentration was doubled. This cycle was repeated, resulting in final concentrations of 960 nM for SUDHL2 and 832 nM for SUDHL6. The entire induction process lasted approximately 6–7 months. After establishment, the resistant cell lines (designated SUDHL2R and SUDHL6R) were routinely maintained in medium containing 110 and 105 nM DOX, respectively, and were cultured without DOX for 1 week prior to experiments to eliminate acute drug effects. To verify the stability of the resistant phenotype, the resistant cells were continuously passaged in drug‐free medium, and their IC50 values were determined at 2 and 5 weeks after drug withdrawal. The results showed that the IC50 values at both time points remained significantly higher than those of the parental cells, with resistance indices (RI = IC50 of resistant cells/IC50 of parental cells) all greater than 5, meeting the accepted criteria for successful establishment of resistant cell lines and indicating that the resistant phenotype was stably maintained after drug removal (Figure S1A–D).

### 2.3 Cell proliferation assay

Cell viability and the IC50 of DOX were assessed using the CCK‑8 assay. Briefly, DLBCL cells after specific treatments were seeded into 96‑well plates at a density of 1 × 10^4^ cells per well in 100 µL of medium and cultured for indicated periods. Subsequently, 10 µL of CCK‑8 reagent was added to each well, and the plates were incubated at 37°C for 1 h in the dark. Absorbance was then measured at 450 nm using a microplate reader.

### 2.4 Cell apoptosis assay

Apoptosis was assessed using the Annexin V‑FITC/PI double‑staining method. Briefly, treated cells were collected, centrifuged at 1000 rpm for 5 min and washed twice with ice‑cold PBS. The cell pellets were then resuspended in 500 µL of Annexin V Binding Buffer, followed by the addition of 5 µL Annexin V‑FITC and 5 µL PI solution, and incubated at room temperature for 15 min in the dark. Apoptotic cells were analysed using a BD FACS Aria III flow cytometer, and the data were processed with FlowJo 10.8 software to quantify apoptotic populations.

### 2.5 Immunohistochemistry

Immunohistochemistry (IHC) staining was performed according to standard protocols. Stained sections were independently evaluated by two investigators. PRMT7 positivity was evaluated based on a combined score of staining intensity and percentage of positive cells.^18^

### 2.6 Co‐immunoprecipitation (Co‐IP)

Treated cells were collected and lysed in pre‑chilled Western/IP lysis buffer (Beyotime) on ice for 30 min. The lysates were then centrifuged at 12 000 rpm for 10 min at 4°C to collect the supernatant, which was subsequently incubated with specific primary antibodies and Protein A/G magnetic beads (Thermo Fisher Scientific) overnight at 4°C. On the following day, the beads were collected by centrifugation, washed thoroughly with Western/IP lysis buffer and resuspended in 1× SDS‑PAGE loading buffer, followed by boiling for 10 min. The precipitated proteins were then analysed by Western blotting. For immunoprecipitation of exogenous tagged proteins, BeyoMag™ Anti‑GST Magnetic Beads, Anti‑Flag Magnetic Beads and Anti‑HA Magnetic Beads (all from Beyotime) were used accordingly.

### 2.7 CRISPR/Cas9 technique

The sgRNA sequences targeting PRMT7 were designed and purchased from Merck. The sgRNA was cloned into the lentiCRISPR v2 vector. The sequence of sgRNA‐PRMT7‐1 is AACATCATAGAGAAACGGC, and the sequence of sgRNA‐PRMT7‐2 is GGCTTCGACGTGCACATCA.

### 2.8 Cell transfection

DLBCL cells in the logarithmic growth phase were transduced with PRMT7‑expressing lentivirus in medium containing 1×HitransG. After incubation at 37°C with 5% CO_2_ for 24 h, the medium was replaced with fresh complete medium, and cells were cultured for an additional 48 h. Stable transfectants were then selected by puromycin treatment for 6 days.

### 2.9 Western blotting

After collection and PBS washing, cells were lysed in RIPA buffer containing protease inhibitors on ice for 30 min. The lysates were centrifuged at 12 000×*g* for 10 min at 4°C, and the supernatants were collected. Protein samples were mixed with 5× SDS loading buffer and denatured by boiling at 95°C for 10 min. The proteins were resolved by SDS‑PAGE and then transferred onto PVDF membranes. The membranes were blocked with 5% skim milk at room temperature for 1 h, followed by incubation with primary antibodies at 4°C overnight. After washing, the membranes were incubated with appropriate secondary antibodies at room temperature for 1 h. Protein signals were visualised using ECL chemiluminescent reagent and detected with the Bio‑Rad Image Lab system.

### 2.10 In vitro methylation assay

Recombinant His‑tagged FOXK1‑(Wild Type) WT and FOXK1‑R191K mutant were expressed in E. coli BL21 cells and purified using a His‑tag protein purification kit (Beyotime, Shanghai, China). Recombinant GST‑tagged PRMT7 (wild‑type or LDIG mutant) was expressed in HEK293T cells and purified with BeyoMag™ Anti‑GST magnetic beads (Beyotime). The purified His‑FOXK1 proteins were incubated with purified GST‑PRMT7 or GST‑LDIG in the presence of 100 µM S‑adenosyl‑L‑methionine (Solarbio, Beijing, China) at 30°C for 1 h. The reactions were terminated by addition of 5× SDS‑PAGE sample buffer, and the products were resolved by SDS‑PAGE, followed by Western blotting. In vitro methylation assays for FOXK2 were performed under identical conditions.

### 2.11 Quantitative real‐time PCR

Total RNA was extracted using the TRIzol method according to the manufacturer's instructions. RNA concentration and quality were measured using the NanoDrop 2000 (Thermo Fisher Scientific). The extracted RNA was reverse transcribed into cDNA using the HiScript II Q RT SuperMix for qPCR kit (R223‐01; Vazyme) following the manufacturer's protocol. Subsequently, target regions of cDNA were amplified using the HiScript II One‐Step qRT‐PCR SYBR Green kit (Q221‐01; Vazyme). Relative gene expression levels were determined using the 2−ΔΔCt method. The primer sequences are presented in Table 1.

**TABLE 1:** The primer sequence of genes.

Gene	Forward primer	Reverse primer
FOXK1	5ʹ‐CATTACCCCTACTACCGGACG‐3ʹ	5ʹ‐GTAACGGTTCAAAGAGAGGTTGT‐3′
FOXK2	5ʹ‐GGACTAAACGGCACCAGAAA‐3ʹ	5ʹ‐CCATCCAAGCCTCCATCATA‐3′
AXIN2	5ʹ‐GTCCAGCAAAACTCTGAGGG‐3ʹ	5ʹ‐CTGGTGCAAAGACATAGCCA‐3ʹ
CCND1	5ʹ‐GACCTTCGTTGCCCTCTGT‐3ʹ	5ʹ‐TGAGGCGGTAGTAGGACAGG‐3ʹ
MYC	5′‐GCCTCAGAGTGCATCGAC‐3′	5ʹ‐TCCACAGAAACAACATCG‐3′
β‐Tubulin	5′‐ACCAACCTACGGGGATCTGAA‐3′	5ʹ‐TTGACTGCCAACTTGCGGA‐3′
DVL2	5′‐GAGGAAGAGACTCCCTACCTG‐3′	5ʹ‐CGGGCGTTGTCATCTGAAAT‐3

### 2.12 Immunofluorescence staining

Logarithmically growing cells with good viability were collected by centrifugation and gently resuspended in 20 µL of sterile PBS to adjust the cell density to approximately 2 × 10^5^ cells per coverslip. The cell suspension was then dropped onto poly‐L‐lysine pre‐coated coverslips and evenly spread across the central area using a 20 µL micropipette tip held at an oblique angle, with multiple ‘Z’‐shaped strokes. Thereafter, a hydrophobic barrier was drawn around the smeared area using an immunohistochemistry pen (PAP pen; Servicebio) to confine the subsequent reaction solutions. Cells were fixed with 4% paraformaldehyde for 10 min, washed three times with PBS and permeabilised with .5% Triton X‑100 for 5 min, followed by another three PBS washes. After blocking with 1% BSA for 1 h, the slides were incubated overnight at 4°C in the dark with an anti‑DVL2 primary antibody. The following day, after PBS washing, the cells were incubated with a fluorescent secondary antibody for 1 h at room temperature in the dark. After additional PBS washes, the nuclei were counterstained with DAPI‑containing mounting medium for 30 min in the dark. The subcellular distribution of DVL2 was examined under a confocal laser scanning microscope, and fluorescence intensities in the nucleus and cytoplasm were quantified using ImageJ software.

### 2.13 Nucleus and cytoplasm separation

Nuclear and cytoplasmic proteins were extracted using a Nuclear and Cytoplasmic Protein Extraction Kit (P0028; Beyotime). Briefly, 20 µL of cell pellet was resuspended in 200 µL of ice‑cold Cytoplasmic Extraction Reagent A containing PMSF, incubated on ice for 15 min and then mixed with 10 µL of Cytoplasmic Extraction Reagent B for 1 min on ice. After centrifugation at 16 000×*g* for 5 min at 4°C, the supernatant containing cytoplasmic proteins was collected. The remaining pellet was resuspended in 50 µL of Nuclear Extraction Reagent supplemented with PMSF, incubated on ice for 30 min and centrifuged at 16 000×g for 10 min at 4°C. The resulting supernatant containing nuclear proteins was collected for further analysis.

### 2.14 Animal model establishment

In this study, 4‐week‐old male NOD‐SCID mice (purchased from Beijing Vital River Laboratory Animal Technology Co., Ltd.) were housed in SPF‐grade animal facilities. A total of 1 × 10^7^ DLBCL cells were subcutaneously injected into the right dorsal flank of mice to establish xenograft tumour models. When tumour volume reached 50 mm^3^, DOX or cell penetrating peptide (CPPtat‐1) was administered via tail vein injection. Tumour volume (*V* = *L* × *W* ^2^/2) was monitored every 3 days. After 27 days, all mice were euthanised, and xenografts were collected for photography and weighing. All experimental procedures were approved by the Animal Ethics Committee of Tongji Medical College, Huazhong University of Science and Technology and strictly complied with laboratory animal welfare ethics guidelines.

### 2.15 Antibodies and chemicals

The following antibodies were used in this study: anti‐FOXK1 (Abclonal; A27583), anti‐FOXK2 (Cell Signalling, 28712), anti‐mono‐Methyl Arginine (Cell Signalling; 8015), PRMT7 (Abclonal; A20975), anti‐HA (ProteinTech; 51064‐2‐AP), anti‐Flag (ProteinTech; 20543‐1‐AP), anti‐GST (ProteinTech; 10000‐0‐AP), anti‐His (ProteinTech; 66005‐1‐Ig), anti‐DVL2 (ProteinTech; 12037‐1‐AP), anti‐HDAC1 (Proteintech Cat; 10197‐1‐AP), anti‐β‐tubulin (Abclonal; A12289), anti‐AXIN2 (ProteinTech; 20540‐1‐AP), anti‐c‐Myc (ProteinTech; 10828‐1‐AP), anti‐CCND1 (ProteinTech; 60186‐1‐Ig). The following chemicals were used: DOX (Med Chem Express; HY‐15142), PRMT7 inhibitor (Med Chem Express; SGC3027).

### 2.16 Statistical analysis

All statistical analyses were performed using GraphPad Prism 8.0 software and SPSS Statistics 24.0. For xenograft experiments evaluating the biological function of PRMT7, mice were randomly assigned to different groups before tumour cell implantation and subsequently subcutaneously inoculated with control, PRMT7‐knockdown or PRMT7‐overexpressing DLBCL cells. For drug treatment experiments, mice bearing established tumours were randomly allocated into different treatment groups after tumour volumes reached 50 mm^3^. The investigators were not blinded to allocation during experiments and outcome assessment in the xenograft studies. The sample size was determined based on our pilot experiments, previous experience with the xenograft model and comparable published studies.^6^, ^19^, ^20^

Comparisons between two groups were performed using *t*‐test, whereas comparisons among three or more groups were performed using one‐way ANOVA with Tukey's post hoc test. For analyses involving multiple variables, we employed two‐way ANOVA with Bonferroni's post hoc tests for multiple comparisons. As the IHC PRMT7 staining scores are ordinal data, comparisons between the two groups were performed using the non‐parametric Mann–Whitney *U* test. Overall survival (OS) was estimated using the Kaplan–Meier method, and differences between groups were compared using the log‐rank test. For categorical variables, comparisons between the two groups were performed using the chi‐square test. Data were presented as mean ± standard deviation (mean ± SD). A *p* value of less than .05 was considered statistically significant. The following notation was used to indicate significance levels: ns (not significant), *p < .05, **p < .01, ***p < .001 and ****p < .0001.

## 3 RESULTS

### 3.1 Aberrant PRMT7 expression promotes DLBCL cell proliferation

**FIGURE 1:**
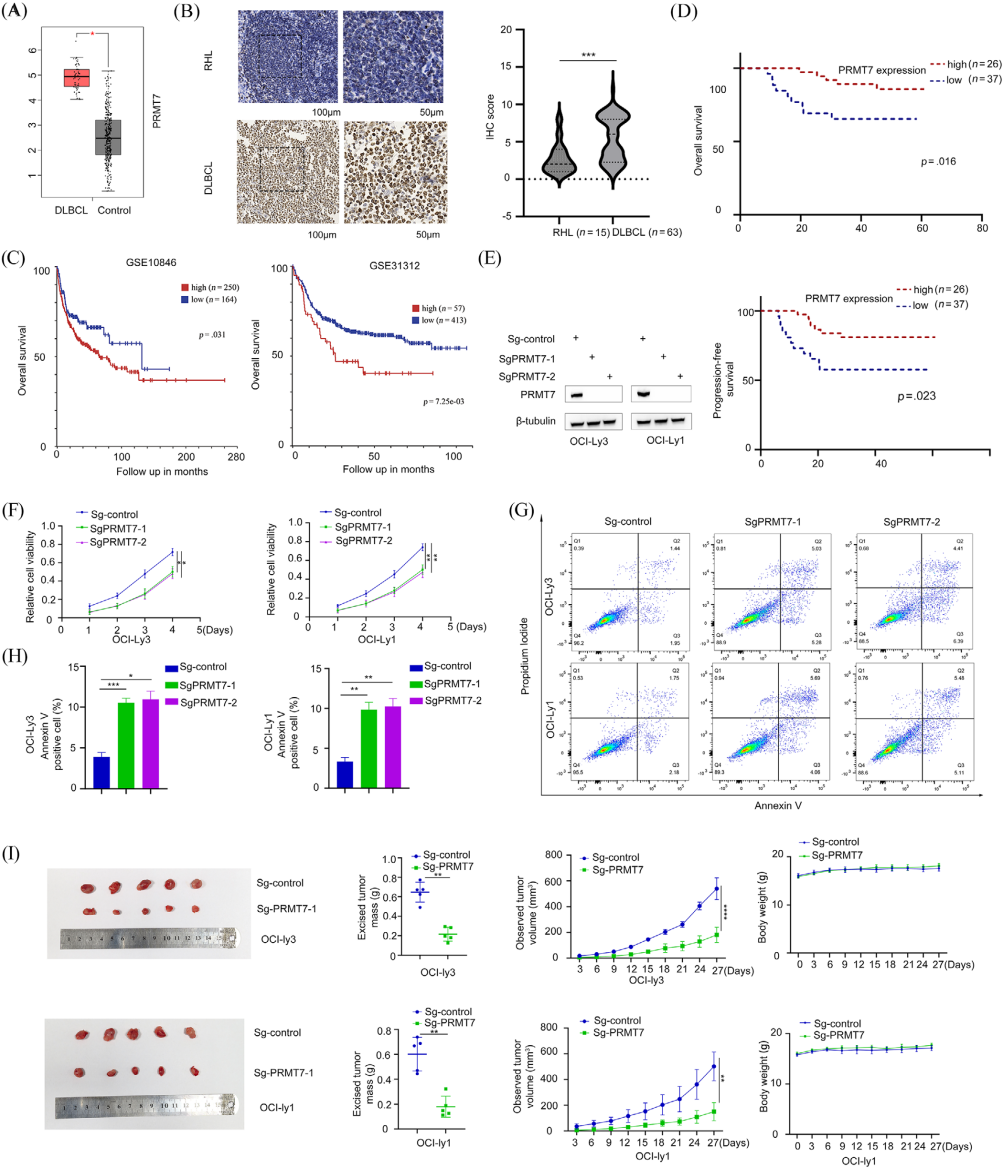
Aberrant PRMT7 expression promotes DLBCL cell proliferation. (A) Expression levels of PRMT7 in DLBCL patients and healthy control donors, data sourced from the GEPIA database. (B) Representative IHC images of PRMT7 staining in reactive lymphoid hyperplasia (RHL) and DLBCL tissues (scale bars: 100 and 50 µm). The violin plot on the right quantifies the IHC staining scores (Mann–Whitney *U* test). (C) Overall survival curves for DLBCL patients with high versus low PRMT7 expression were plotted using data from the GSE10846 and GSE31312 datasets from the GEO database. (D) Kaplan–Meier survival analysis was performed to evaluate the prognostic significance of PRMT7 expression (high vs. low) for both OS and PFS in our cohort, with statistical significance determined by the Log‐rank test. (E) PRMT7 expression levels detected by Western blotting after knockout of PRMT7 in OCI‐Ly3 and OCI‐Ly1 cell lines. Data are representative of three independent experiments. (F) Cell proliferation detected by CCK8 assay after knockout of PRMT7 in OCI‐Ly3 and OCI‐Ly1 cell lines. The experiments were repeated three independent times, two‐way ANOVA followed by followed by Bonferroni's post hoc test. (G and H) Apoptosis percentage detected by flow cytometry after knockout of PRMT7 in OCI‐Ly3 and OCI‐Ly1 cell lines. The experiments were repeated three independent times. One‐way ANOVA followed by Tukey's multiple comparison test (H). (I) PRMT7‐knockout OCI‐Ly3 and OCI‐Ly1 cells were injected into the left flank of nude mice, and tumour tissues were collected after 27 days: photographed, weighed (*n* = 5 mice), data are representative of three independent experiments. Statistical analyses were performed using two‐way ANOVA followed by Bonferroni's post hoc test for tumour volume curves and body weight, and one‐way ANOVA followed by Tukey's multiple comparison test for excised tumour weights.

First, we assessed PRMT7 expression in DLBCL. Bioinformatics analysis using the GEPIA database showed that PRMT7 was significantly elevated in DLBCL patients compared with normal controls (Figure 1A). This upregulation was consistently observed in our DLBCL patient cohort (Figure 1B) and across multiple DLBCL cell lines (Figure S2A,B). Survival analysis based on GEO datasets revealed that high PRMT7 expression was significantly associated with shorter OS (Figure 1C). In our follow‑up cohort, baseline clinicopathological characteristics are summarised in Table 2. Survival analysis further indicated that high PRMT7 expression correlated with worse OS and progression‑free survival (PFS) (Figure 1D).

**TABLE 2:** The characteristics of DLBCL patients.

	PRMT7 expression		
	Low (*n* = 37)	High (*n* = 26)	*p* Value
Age (years)			.889
>60 years	12	8	
≤60 years	25	18	
Gender			.620
Male	19	15	
Female	18	11	
IPI score			.555
0–2	20	16	
3–5	17	10	
Disease stage			.966
I–II	13	9	
III–IV	24	17	
ECOG PS			.558
≤1	26	20	
>1	11	6	
Extranodal site(s)			.264
1 or 0	22	19	
>1	15	7	
LDH			.536
≤ULN (270 µmol/L)	17	14	
>ULN (270 µmol/L)	20	12	
Hans classification			.941
GCB	16	11	
Non‐GCB	21	15	

To further clarify the functional role of PRMT7 in DLBCL, we established PRMT7‑knockout DLBCL cell lines (Figure 1E). CCK‑8 and apoptosis assays showed that PRMT7 knockout significantly inhibited cell proliferation and promoted apoptosis in both ABC‑ and GCB‑subtype DLBCL cells in vitro and also suppressed tumour growth in vivo (Figure 1F–I). Conversely, PRMT7 overexpression consistently enhanced the progression of both subtypes in vitro and in vivo (Figure S3A–D). Collectively, these findings demonstrate that elevated PRMT7 expression promotes DLBCL tumour progression.

### 3.2 Upregulated PRMT7 induces chemoresistance to DOX in DLBCL cells

**FIGURE 2:**
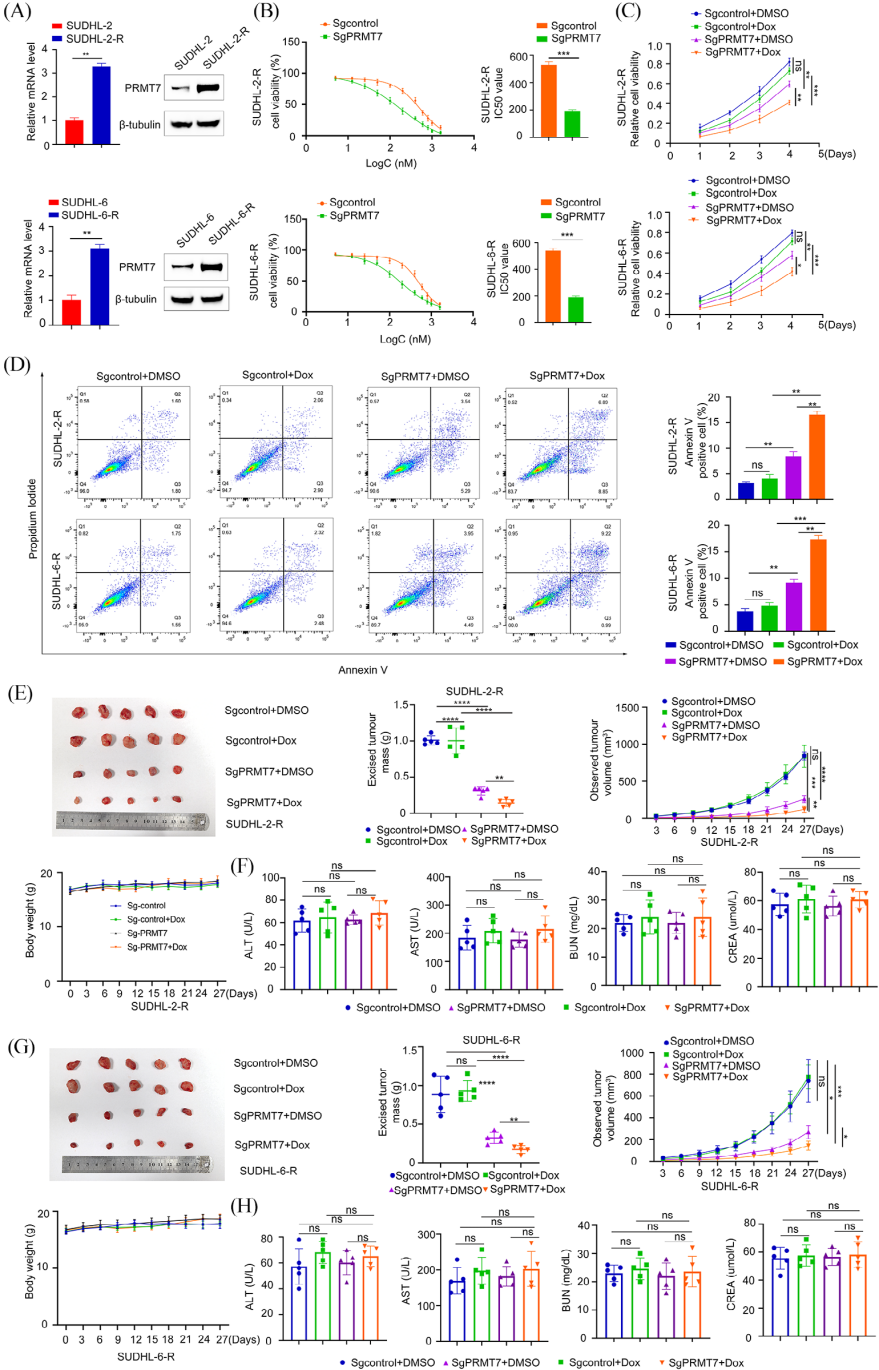
Upregulated PRMT7 induces chemoresistance to doxorubicin in DLBCL cells. (A) The expression levels of PRMT7 in SUDHL‐2/SUDHL‐2R and SUDHL‐6/SUDHL‐6R cells were detected by qRT‐PCR and Western blotting. The experiments were repeated three independent times. Student's *t*‐test. (B) After PRMT7 knockout in SUDHL‐2R and SUDHL‐6R cells, IC50 of doxorubicin was determined by CCK‐8 assay. The experiments were repeated three independent times. Student's *t*‐test. (C) PRMT7 was knocked out, and cells were treated with and without doxorubicin. Cell proliferation of SUDHL‐2R and SUDHL‐6R cells was assessed by CCK‐8 assay. The experiments were repeated three independent times. Two‐way ANOVA followed by followed by Bonferroni's post hoc test. (D) PRMT7 was knocked out, and cells were treated with doxorubicin. Percentage of apoptosis in SUDHL‐2R and SUDHL‐6R cells was determined by flow cytometry. The experiments were repeated three independent times. One‐way ANOVA followed by Tukey's multiple comparison test. (E and G) PRMT7‐knockout SUDHL‐2R and SUDHL‐6R cells were injected into the left abdominal wall of nude mice. When the tumour volume reached 50 mm^3^, doxorubicin was injected every 3 days. Tumour volume and body weight of mice were measured every 3 days. After 27 days, tumour tissues were collected for photography and weighing. *n* = 5 mice, data are representative of three independent experiments. Statistical analyses were performed using two‐way ANOVA followed by Bonferroni's post hoc tests for tumour volume curves and body weight, and one‐way ANOVA followed by Tukey's multiple comparison test for excised tumour weights. (F and H) Serum biochemical markers of liver and kidney function were assessed on Day 27. Liver function indicators, including alanine aminotransferase (ALT) and aspartate aminotransferase (AST). Kidney function indicators, including blood urea nitrogen (BUN) and creatinine (CREA). The data are representative of three independent experiments. One‐way ANOVA followed by Tukey's multiple comparison test.

Beyond its role in tumour progression, we further investigated whether PRMT7 contributes to DOX resistance in DLBCL. DOX is a cornerstone anthracycline for DLBCL treatment, yet its clinical efficacy is frequently hampered by acquired resistance.^4^ To evaluate the impact of PRMT7 on chemosensitivity, we overexpressed or knocked out PRMT7 in DLBCL cell lines. PRMT7 overexpression markedly reduced DOX sensitivity, whereas PRMT7 knockout restored drug responsiveness (Figures S4A,B and S5A–C). We then established DOX‑resistant DLBCL sublines (SUDHL‑2R and SUDHL‑6R) and observed a significant upregulation of PRMT7 expression in both resistant lines (Figure 2A,B). Notably, PRMT7 knockout resensitised these resistant cells to DOX (Figure 2C).

Functional assays further demonstrated that PRMT7 knockout significantly inhibited proliferation and promoted apoptosis in the resistant cells (Figure 2D). In vivo, PRMT7 depletion markedly enhanced the antitumour efficacy of DOX against resistant xenografts, as evidenced by reduced tumour growth (Figure 2E,G). No significant body weight changes were observed across groups during treatment. Furthermore, serum levels of liver function markers (alanine aminotransferase [ALT], aspartate aminotransferase [AST]) and renal function markers (urea nitrogen [BUN] and creatinine [CREA]) showed no statistically significant differences among groups, indicating an acceptable safety profile (Figure 2F,H). Collectively, these results indicate that PRMT7 knockout effectively enhances the anti‐lymphoma efficacy of DOX without causing obvious toxicities. Collectively, these findings demonstrate that upregulation of PRMT7 expression promotes DOX resistance in DLBCL cells, whereas inhibiting PRMT7 re‐sensitises the cells to DOX.

### 3.3 FOXK1/2 are substrate proteins of PRMT7 and are methylated by PRMT7

**FIGURE 3:**
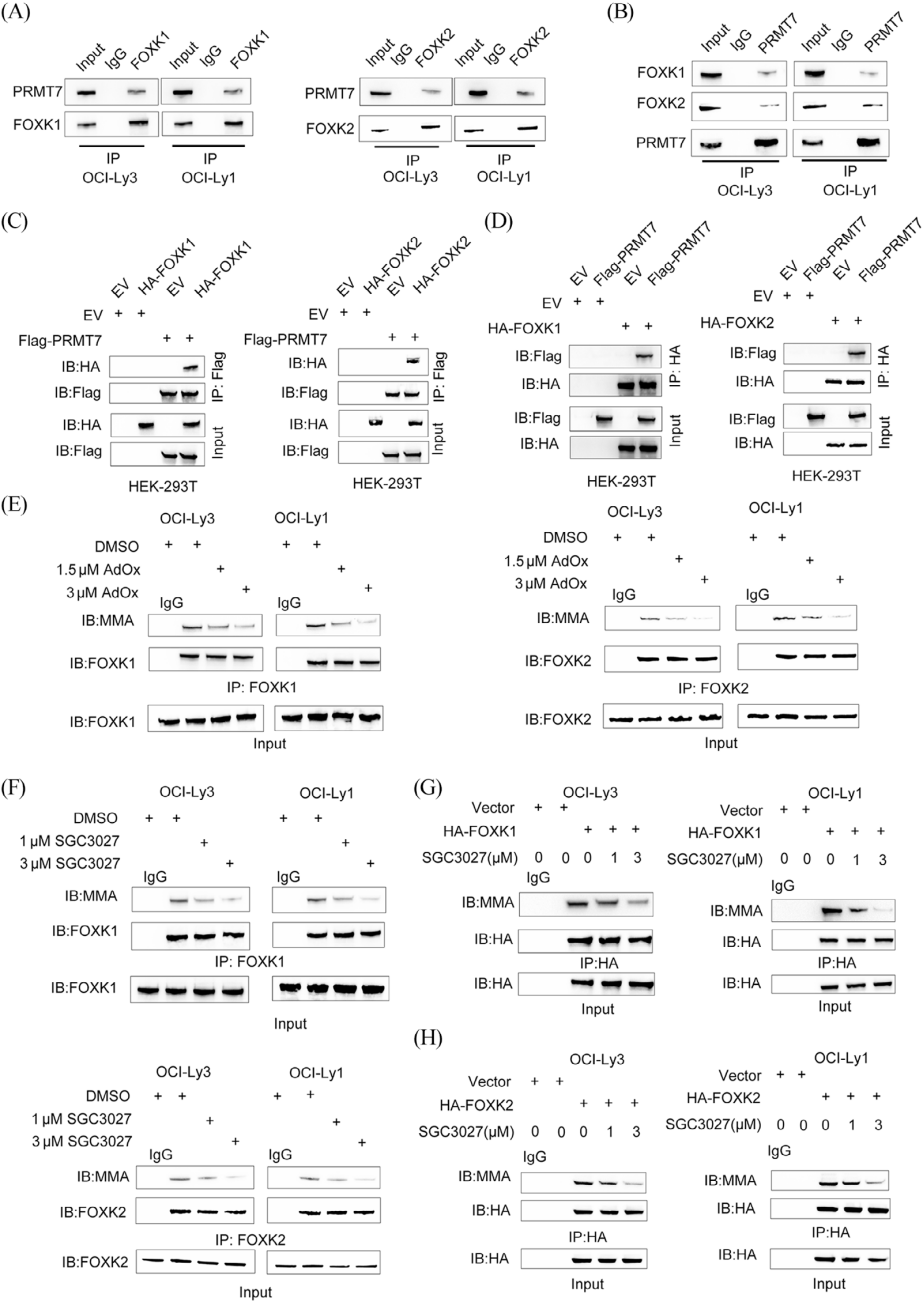
FOXK1/2 are substrate proteins of PRMT7 and are methylated by PRMT7. (A and B) Western blotting analysis of Co‐IP for endogenous PRMT7 and FOXK1, as well as PRMT7 and FOXK2, in OCI‐Ly3 and OCI‐Ly1 cells. The data are representative of three independent experiments. (C and D) The indicated plasmids were transfected into 293T cells. Co‐IP was performed to detect interactions between exogenous Flag‐PRMT7 and HA‐FOXK1 or Flag‐PRMT7 and HA‐FOXK2. The data are representative of three independent experiments. (E) OCI‐Ly3 and OCI‐Ly1 cells were treated with AdOx. IP was performed to detect the monomethylation level of FOXK1 or FOXK2. The data are representative of three independent experiments. (F) OCI‐Ly3 and OCI‐Ly1 cells were treated with SGC3027. IP was performed to detect the MMA level of FOXK1 or FOXK2. The data are representative of three independent experiments. (G) HA‐FOXK1 plasmid was transfected into OCI‐Ly3 and OCI‐Ly1 cells, followed by treatment with SGC3027. IP was performed to detect the MMA level of FOXK1. The data are representative of three independent experiments. (H) HA‐FOXK2 plasmid was transfected into OCI‐Ly3 and OCI‐Ly1 cells, followed by treatment with SGC3027. IP was performed to detect the MMA level of FOXK2. The data are representative of three independent experiments.

PRMT7 is an arginine methyltransferase that modulates substrate protein function through methylation^11^; however, the molecular mechanism by which it mediates DOX resistance in DLBCL remains largely unknown. A recent methylomics study identified FOXK1 and FOXK2 as potential PRMT7 substrates.^21^ As key members of the FOXK family, these transcription factors are involved in diverse biological processes, including glucose metabolism^15^ and DNA damage repair.^14^ To determine whether PRMT7 interacts with FOXK1/2, we performed Co‑IP assays, which confirmed direct binding of PRMT7 to both FOXK1 and FOXK2 (Figure 3A–D). To further define the subcellular compartment of this interaction, we generated FOXK1 and FOXK2 mutants lacking the nuclear localisation signal (NLS), which were consequently retained in the cytoplasm (Figure S6A). Co‑IP analysis showed that although FOXK1ΔNLS and FOXK2ΔNLS failed to enter the nucleus efficiently, they retained robust binding to PRMT7, with binding affinities comparable to those of FOXK1‐WT and FOXK2‐WT, indicating that this interaction occurs in the cytoplasm (Figure S6B).

We next investigated whether PRMT7 regulates FOXK1/2 through post‑translational methylation. Our results confirmed that FOXK1 and FOXK2 are methylated and that the non‑specific methyltransferase inhibitor Adox suppressed their methylation in a dose‑dependent manner (Figure 3E). Similarly, the PRMT7‑specific inhibitor SGC3027 also reduced FOXK1/2 methylation in a dose‑dependent fashion (Figure 3F). Furthermore, PRMT7 inhibition in the context of FOXK1 or FOXK2 overexpression significantly diminished their methylation levels (Figure 3G,H). Collectively, these data demonstrate that PRMT7 interacts with FOXK1/FOXK2 and catalyses their arginine monomethylation.

### 3.4 PRMT7 catalyses methylation of FOXK1 at arginine 191 and FOXK2 at arginine 144

**FIGURE 4:**
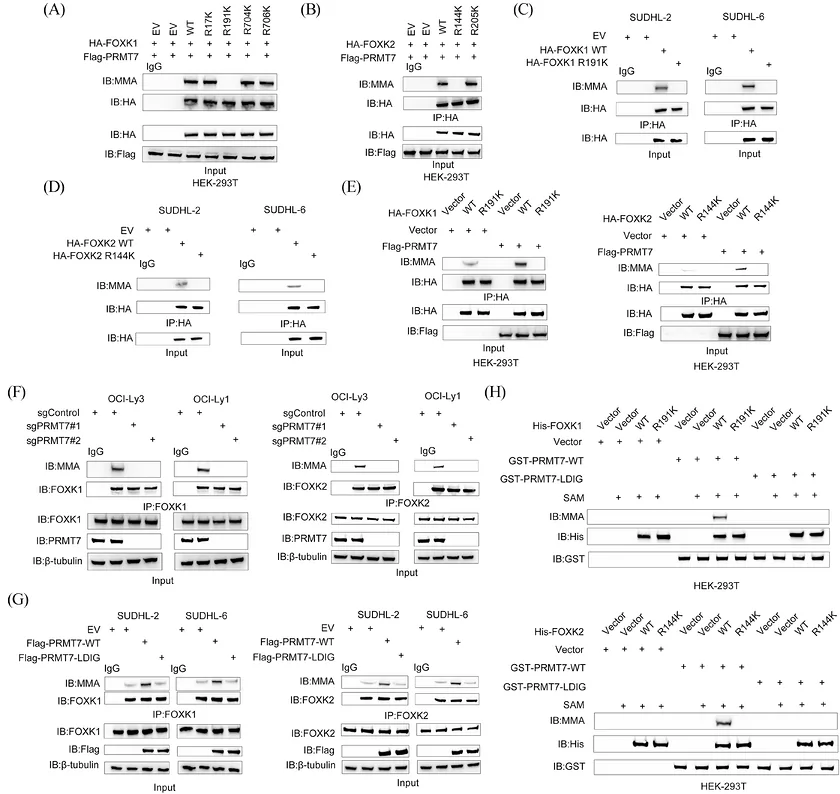
PRMT7 catalyses monomethylation of FOXK1 at arginine 191 and FOXK2 at arginine 144. (A) The indicated plasmids were transfected into 293T cells. The MMA levels of different FOXK1 mutants were measured by IP. The data are representative of three independent experiments. (B) The indicated plasmids were transfected into 293T cells. The MMA levels of different FOXK2 mutants were measured by IP. The data are representative of three independent experiments. (C) The indicated plasmids were transfected into SUDHL‐2 and SUDHL‐6 cells. IP was performed to detect the MMA level of FOXK1. The data are representative of three independent experiments. (D) The indicated plasmids were transfected into SUDHL‐2 and SUDHL‐6 cells. IP was performed to detect the MMA level of FOXK2. The data are representative of three independent experiments. (E) The indicated plasmids were transfected into 293T cells. IP was performed to detect the MMA level of FOXK1 or FOXK2. The data are representative of three independent experiments. (F) After PRMT7 knockout in OCI‐Ly3 and OCI‐Ly1 cells, IP was performed to detect the MMA level of FOXK1 or FOXK2. The data are representative of three independent experiments. (G) The indicated plasmids were transfected into SUDHL‐2 and SUDHL‐6 cells. IP was performed to detect the MMA level of FOXK1 or FOXK2. The data are representative of three independent experiments. (H) In vitro methylation assay was performed to detect the MMA level of FOXK1 or FOXK2. The data are representative of three independent experiments.

As noted above, methylomics analysis identified FOXK1 and FOXK2 as being monomethylated at R191 and R144, respectively. To validate these sites, we employed methylation‑specific prediction software (Figure S7A,B) and generated the corresponding Arg‑to‑Lys point mutants. Western blotting revealed that the R191K mutation specifically abolished FOXK1 methylation, whereas the R144K mutation exerted an identical effect on FOXK2 (Figure 4A,B). In SUDHL‐2 and SUDHL‐6 cell lines, overexpression of wild‐type FOXK1 (FOXK1‐WT) significantly presented monomethylation levels of FOXK1, while the FOXK1‐R191K mutant showed no such effect (Figure 4C); the FOXK2‑WT and FOXK2‑R144K mutants exhibited a similar pattern (Figure 4D). Notably, neither PRMT7 overexpression nor PRMT7 inhibitor treatment affected the monomethylation levels of these mutants, further confirming that R191 and R144 are the critical sites for FOXK1 and FOXK2, respectively (Figures 4E and S7D–E).

Cross‑species sequence alignment showed that FOXK1‑R191 and FOXK2‑R144 are highly evolutionarily conserved, and interestingly, their surrounding sequences exhibit substantial homology (Figure S7C). To determine the functional impact of PRMT7 on FOXK1/2 monomethylation, we found that PRMT7 knockout significantly reduced their monomethylation levels (Figure 4F), whereas PRMT7‑WT enhanced them; the catalytically inactive mutant PRMT7‑LDIG failed to do so (Figure 4G). In vitro methylation assays further confirmed that both PRMT7 catalytic activity and the R191 residue of FOXK1 are essential for FOXK1 monomethylation; similarly, PRMT7 activity and the R144 residue are required for FOXK2 monomethylation (Figure 4H). Moreover, no obvious dimethylation was detected on either FOXK1 or FOXK2 (Figure S8). Collectively, these data demonstrate that PRMT7 catalyses monomethylation of FOXK1 at arginine 191 and FOXK2 at arginine 144.

### 3.5 Methylation of FOXK1 and FOXK2 promotes their binding to DVL2 and facilitates DVL2 nuclear translocation

**FIGURE 5:**
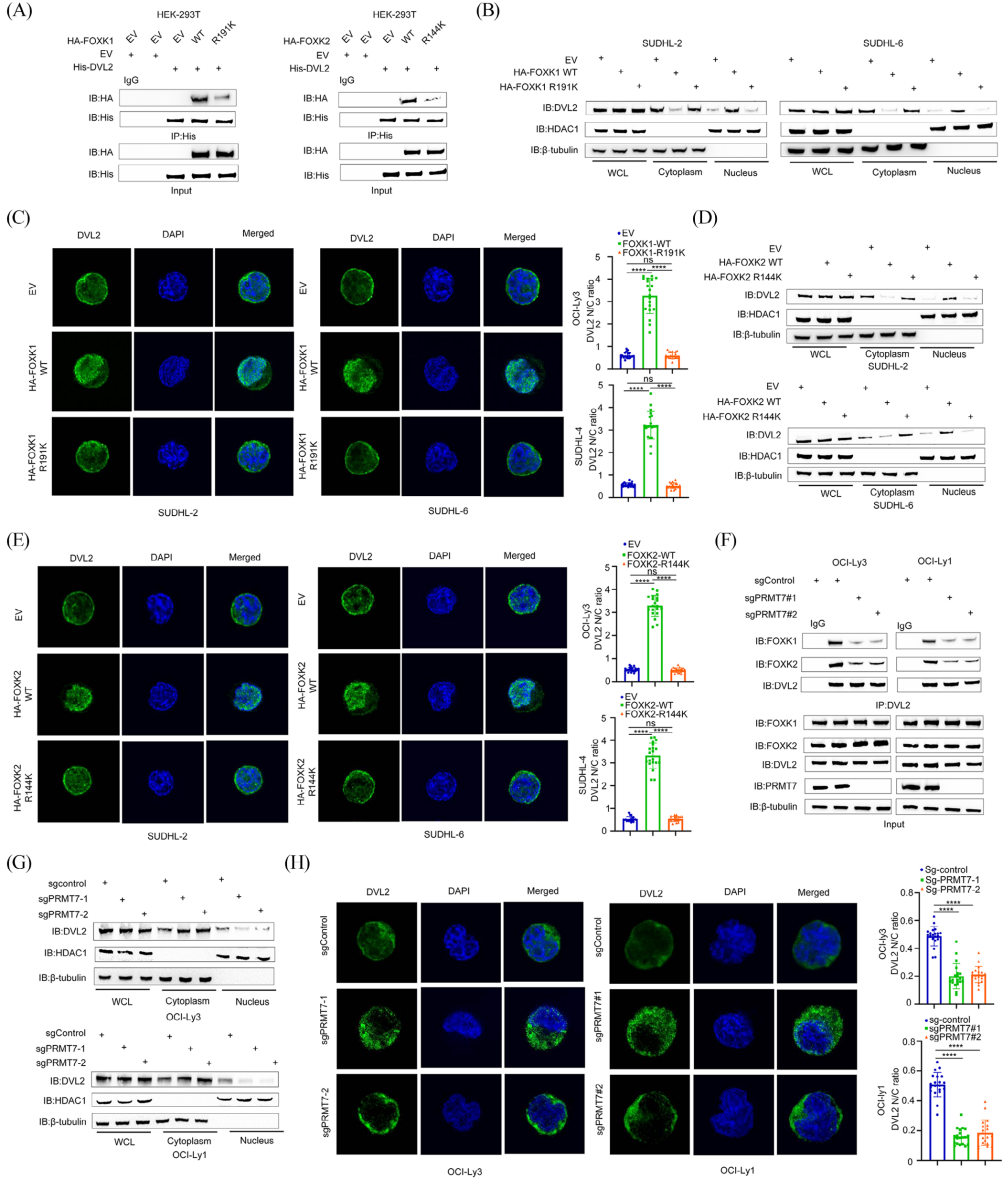
Methylation of FOXK1 and FOXK2 promotes their binding to DVL2 and facilitates DVL2 nuclear translocation. (A)The indicated plasmids were transfected into 293T cells, and Co‐IP was performed to detect the interaction between FOXK1 or FOXK2 and DVL2. The data are representative of three independent experiments. (B and C) SUDHL‐2 and SUDHL‐6 cells were transfected with HA‐FOXK1‐WT or HA‐FOXK1‐R191K plasmid. Nuclear and cytoplasmic fractionation was performed to detect the localisation of DVL2. The indicated cells were transferred to slides for immunofluorescence. Representative images and quantification of 20 cells are in the figure (C). Scale bar: 10 µm. The experiments were repeated three independent times. One‐way ANOVA followed by Tukey's multiple comparison test. (D and E) SUDHL‐2 and SUDHL‐6 cells were transfected with HA‐FOXK2‐WT or HA‐FOXK2‐R144K plasmid. Nuclear and cytoplasmic fractionation was performed to detect the localisation of DVL2 (D). The indicated cells were transferred to slides for immunofluorescence. Representative images and quantification of 20 cells are in the figure (E). Scale bar: 10 µm. The experiments were repeated three independent times. One‐way ANOVA followed by Tukey's multiple comparison test. (F) After PRMT7 knockout in OCI‐Ly3 and OCI‐Ly1 cells, Co‐IP was performed to detect the interaction between FOXK1 or FOXK2 and DVL2; The experiments were repeated three independent times. (G and H) After PRMT7 knockout in OCI‐Ly3 and OCI‐Ly1 cells, nuclear and cytoplasmic fractionation was performed to detect the localisation of DVL2 (G). The indicated cells were transferred to slides for immunofluorescence. Representative images and quantification of 20 cells are in the figure (H). Scale bar: 10 µm. The experiments were repeated three independent times. One‐way ANOVA followed by Tukey's multiple comparison test.

To investigate the functional significance of FOXK1/FOXK2 methylation, we first examined whether PRMT7 regulates the expression of FOXK1/FOXK2. Neither PRMT7 knockout or overexpression nor manipulation of its catalytic activity significantly altered the mRNA or protein levels of FOXK1/FOXK2 (Figure S9A–I), suggesting that PRMT7 does not regulate FOXK1/FOXK2 at the expression level. We therefore hypothesised that PRMT7 may modulate FOXK1/FOXK2 function through arginine methylation. Notably, the PRMT7‐mediated methylation sites, R191 in FOXK1 and R144 in FOXK2, are located in close proximity to their FHA domains, which have previously been reported to mediate the interaction of FOXK1/FOXK2 with DVL2 and facilitate DVL2 nuclear translocation.^22^ We therefore investigated whether methylation of FOXK1/FOXK2 affects their interaction with DVL2 and, consequently, DVL2 nuclear localisation. Co‐IP assays showed that WT‐FOXK1/FOXK2 exhibited robust interactions with DVL2, whereas the methylation‐deficient mutants FOXK1‐R191K and FOXK2‐R144K displayed markedly reduced DVL2 binding (Figure 5A), indicating that methylation at R191 and R144 is critical for the association of FOXK1/FOXK2 with DVL2.

Given the established role of FOXK1/FOXK2 in promoting DVL2 nuclear translocation, we next examined whether FOXK1/FOXK2 methylation influences the subcellular localisation of DVL2. Consistently, nuclear fractionation and immunofluorescence analyses demonstrated that WT‐FOXK1/FOXK2 promoted DVL2 nuclear accumulation, whereas the methylation‐deficient mutants markedly impaired this effect (Figure 5B–E). Furthermore, PRMT7 knockdown reduced the interaction between FOXK1/FOXK2 and DVL2 (Figure 5F) and concomitantly suppressed DVL2 nuclear accumulation (Figure 5G,H).

To determine whether these effects depend on the catalytic activity of PRMT7, we compared PRMT7‐WT with its catalytically inactive mutant (PRMT7‐LDIG). Overexpression of PRMT7‐WT enhanced the interaction between FOXK1/FOXK2 and DVL2 (Figure S10A) and promoted DVL2 nuclear localisation, whereas PRMT7‐LDIG failed to elicit these effects (Figure S10B,C). Importantly, PRMT7 knockout did not significantly alter the mRNA or protein levels of DVL2 (Figure S11), excluding the possibility that the observed changes in DVL2 nuclear accumulation resulted from altered DVL2 expression. In addition, methylation‐deficient mutations of FOXK1/FOXK2 did not affect their own subcellular localisation (Figure S12), further supporting a specific role for FOXK1/FOXK2 methylation in regulating DVL2 trafficking rather than FOXK1/FOXK2 localisation. Collectively, these findings demonstrate that PRMT7‐mediated arginine methylation of FOXK1 at R191 and FOXK2 at R144 enhances their interaction with DVL2, thereby promoting DVL2 nuclear localisation.

### 3.6 Methylation of FOXK1 or FOXK2 promotes activation of the Wnt/β‐catenin signalling pathway

**FIGURE 6:**
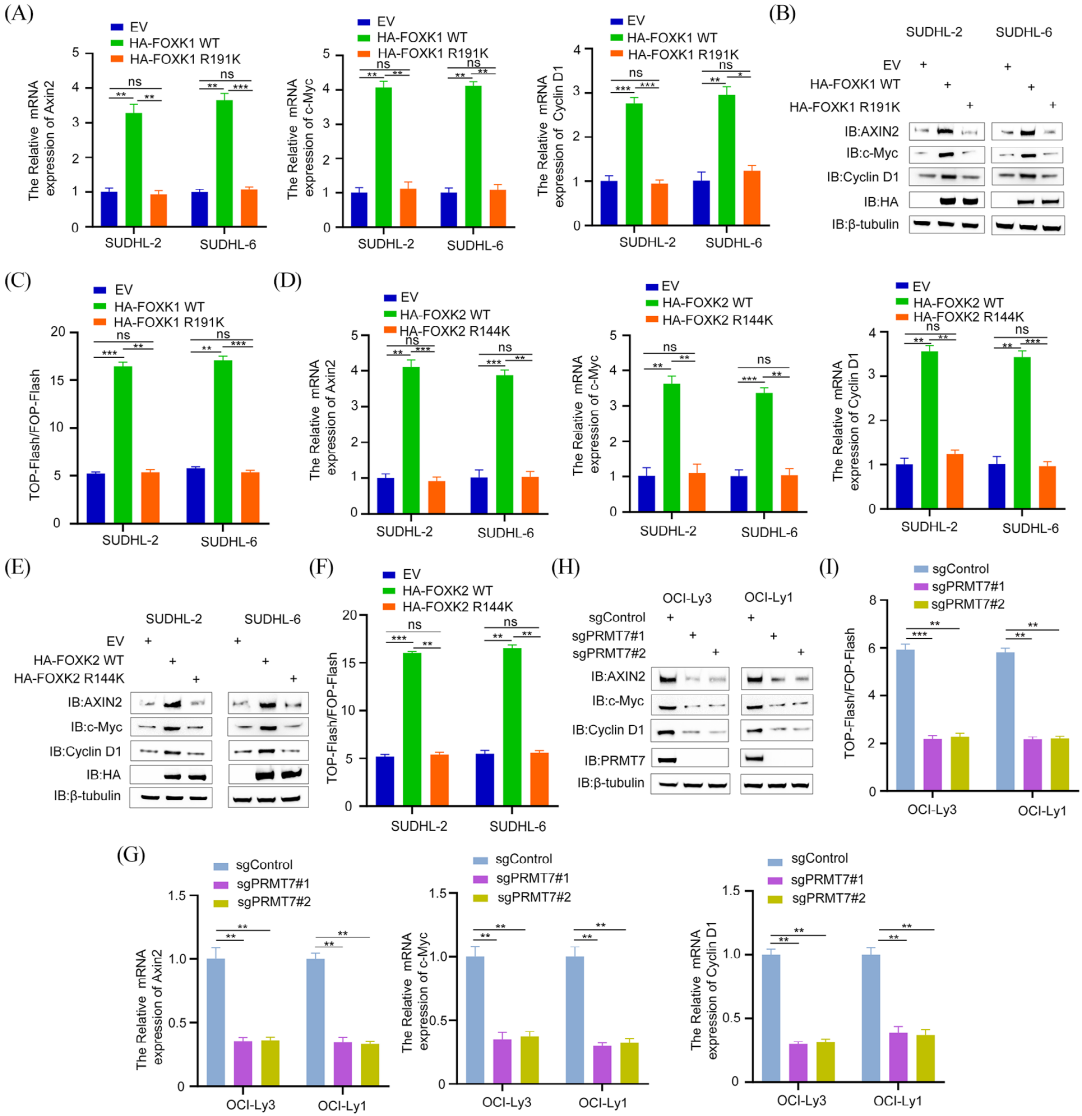
Methylation of FOXK1 or FOXK2 promotes activation of the Wnt/β‐catenin signalling pathway. (A and B) SUDHL‐2 and SUDHL‐6 cells were transfected with HA‐FOXK1‐WT or HA‐FOXK1‐R191K plasmids. mRNA and protein expression levels of Axin2, c‐Myc and cyclin D1 were analysed by qRT‐PCR and Western blotting. The experiments were repeated three independent times. One‐way ANOVA followed by Tukey's multiple comparison test (A). The data are representative of three independent experiments (B). (C) β‐Catenin luciferase reporter assay was conducted in SUDHL‐2 and SUDHL‐6 cells transfected with HA‐FOXK1‐WT or HA‐FOXK1‐R191K plasmid. The experiments were repeated three independent times. One‐way ANOVA followed by Tukey's multiple comparison test. (D and E) SUDHL‐2 and SUDHL‐6 cells were transfected with HA‐FOXK2‐WT or HA‐FOXK2‐R144K plasmids. Expression of Axin2, c‐Myc and cyclin D1 at mRNA and protein levels was determined by qRT‐PCR and Western blotting, respectively. The experiments were repeated three independent times. One‐way ANOVA followed by Tukey's multiple comparison test (D). The data are representative of three independent experiments (E). (F) β‐catenin transcriptional activity was assessed by a luciferase reporter assay in SUDHL‐2 and SUDHL‐6 cells expressing HA‐FOXK2‐WT or HA‐FOXK2‐R144K. The data are representative of three independent experiments, one‐way ANOVA. (G and H) Following PRMT7 knockout in OCI‐Ly3 and OCI‐Ly1 cells, qRT‐PCR and Western blotting were performed to examine Axin2, c‐Myc and cyclin D1 expression. The experiments were repeated three independent times. One‐way ANOVA followed by Tukey's multiple comparison test (G). The data are representative of three independent experiments (H). (I) β‐Catenin‐mediated transcriptional activity was measured by a luciferase reporter assay in PRMT7‐knockout OCI‐Ly3 and OCI‐Ly1 cells. The experiments were repeated three independent times. One‐way ANOVA followed by Tukey's multiple comparison test (I).

FOXK1/FOXK2‐mediated nuclear translocation of DVL2 has been reported to activate Wnt/β‐catenin signalling.^22^ Given our finding that PRMT7‐dependent methylation of FOXK1/FOXK2 promotes DVL2 nuclear localisation, we next investigated whether this modification contributes to Wnt/β‐catenin pathway activation. WT‐FOXK1/FOXK2 markedly increased the mRNA and protein levels of the canonical Wnt target genes AXIN2, c‐Myc and cyclin D1, whereas the methylation‐deficient FOXK1‐R191K and FOXK2‐R144K mutants failed to elicit comparable effects (Figure 6A,B,D,E). Consistently, TOPFLASH reporter assays showed that WT‐ FOXK1/FOXK2 significantly enhanced β‐catenin/TCF‐dependent transcriptional activity, whereas the methylation‐deficient mutants exhibited markedly reduced activity (Figure 6C,F). These findings indicate that methylation of FOXK1/FOXK2 is required for their ability to efficiently activate Wnt/β‐catenin signalling.

We next examined whether PRMT7 regulates Wnt/β‐catenin signalling in a catalytic activity‐dependent manner. PRMT7 knockout significantly reduced the expression of Wnt target genes (Figure 6G,H) and suppressed TOPFLASH reporter activity (Figure 6I). Conversely, overexpression of PRMT7‐WT enhanced Wnt target gene expression and TOPFLASH reporter activity, whereas the catalytically inactive PRMT7 mutant (PRMT7‐LDIG) failed to exert these effects (Figure S13A–C). Together, these results demonstrate that the catalytic activity of PRMT7 is required for efficient activation of Wnt/β‐catenin signalling.

Because canonical Wnt/β‐catenin pathway activation is frequently associated with β‐catenin stabilisation, we next examined total and phosphorylated β‐catenin levels in the context of FOXK1/FOXK2 methylation. Notably, methylation of FOXK1/FOXK2 did not significantly alter either total or phosphorylated β‐catenin levels (Figure S14A–D), suggesting that FOXK1/FOXK2 methylation activates Wnt/β‐catenin signalling through a mechanism independent of β‐catenin stabilisation and accumulation. Previous studies have demonstrated that nuclear DVL can participate in the β‐catenin/TCF transcriptional complex and facilitate Wnt target gene transcription without substantially altering total β‐catenin abundance.^22^, ^23^ Thus, our findings support a model in which PRMT7‐mediated FOXK1/FOXK2 methylation promotes DVL2 nuclear localisation and thereby enhances β‐catenin/TCF‐dependent transcriptional activity rather than increasing β‐catenin abundance.

Finally, to determine whether Wnt/β‐catenin signalling is functionally required for PRMT7‐mediated DOX resistance in DLBCL, we pharmacologically inhibited the pathway and assessed cellular responses to DOX. Wnt/β‐catenin inhibition significantly suppressed cell proliferation and reduced the IC50 of DOX. Importantly, under Wnt/β‐catenin blockade, PRMT7 overexpression failed to restore resistance to DOX (Figure S15A,B), indicating that activation of Wnt/β‐catenin signalling is required for PRMT7‐mediated chemoresistance. Collectively, these findings establish PRMT7‐mediated methylation of FOXK1/FOXK2 as a critical post‐translational mechanism that promotes DVL2 nuclear translocation, thereby activating Wnt/β‐catenin signalling and contributing to DOX resistance in DLBCL.

### 3.7 Methylation of either FOXK1 or FOXK2 enhances DOX chemoresistance in diffuse large B‐cell lymphoma

**FIGURE 7:**
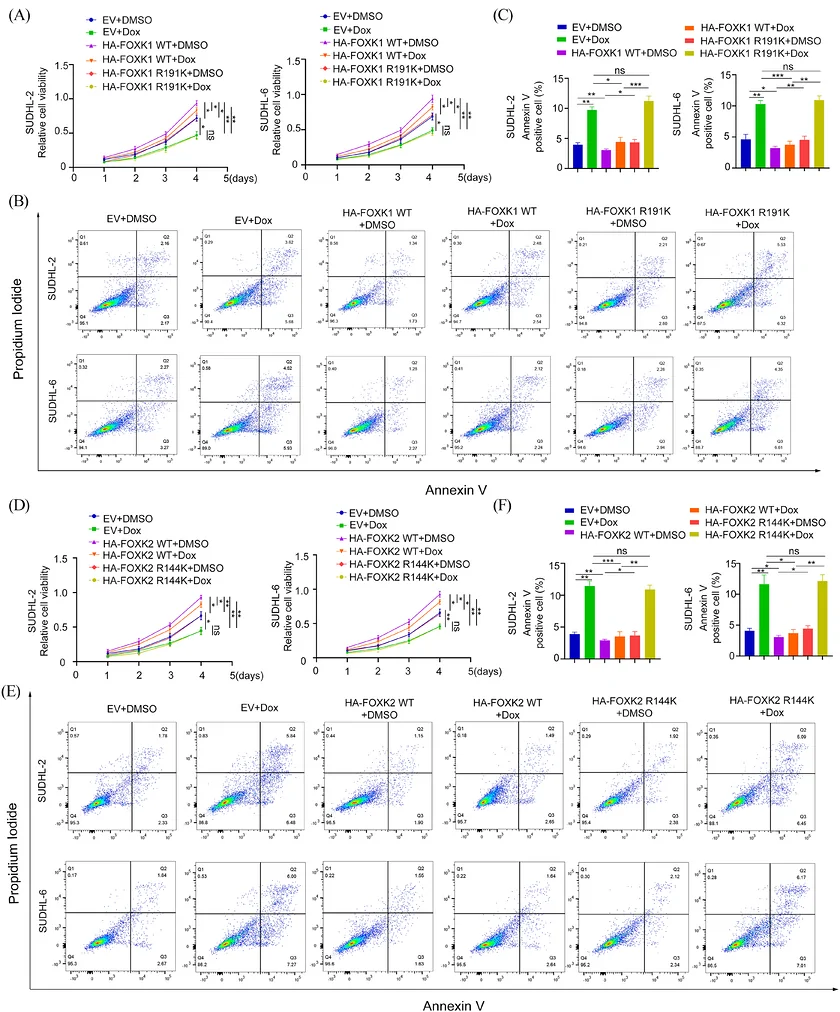
Methylation of either FOXK1 or FOXK2 enhances doxorubicin chemoresistance in diffuse large B‐cell lymphoma. (A) HA‐FOXK1‐WT or HA‐FOXK1‐R191K plasmids were transfected into SUDHL‐2 and SUDHL‐6 cells, followed by doxorubicin treatment. Cell proliferation was assessed using a CCK8 assay. The experiments were repeated three independent times. Two‐way ANOVA followed by Bonferroni's post hoc test (A). (B and C) HA‐FOXK1‐WT or HA‐FOXK1‐R191K plasmids were transfected into SUDHL‐2 and SUDHL‐6 cells, followed by doxorubicin treatment. The proportion of apoptotic cells was detected using an Annexin V‐FITC/PI assay. The experiments were repeated three independent times. One‐way ANOVA followed by Tukey's multiple comparison test (C). The data are representative of three independent experiments (B). (D) HA‐FOXK2‐WT or HA‐FOXK2‐R144K plasmids were transfected into SUDHL‐2 and SUDHL‐6 cells, followed by doxorubicin treatment. Cell proliferation was assessed using a CCK8 assay. The experiments were repeated three independent times. Two‐way ANOVA followed by Bonferroni's post hoc test (D). (E and F) HA‐FOXK2‐WT or HA‐FOXK2‐R144K plasmids were transfected into SUDHL‐2 and SUDHL‐6 cells, followed by doxorubicin treatment. The proportion of apoptotic cells was detected using an Annexin V‐FITC/PI assay. The experiments were repeated three independent times. One‐way ANOVA followed by Tukey's multiple comparison test (F). The data are representative of three independent experiments (E).

Given that PRMT7 promotes DOX resistance in DLBCL and catalyses FOXK1/FOXK2 methylation, we investigated whether FOXK1 or FOXK2 methylation contributes to this chemoresistance. CCK8 assays demonstrated that overexpression of FOXK1‐WT or FOXK2‐WT significantly increased IC_50_ values for DOX, whereas the FOXK1‐R191K or FOXK2‐R144K mutant failed to exhibit this effect (Figure S16A–D). Furthermore, CCK‐8 proliferation and apoptosis assays revealed that FOXK1 methylation conferred DOX resistance in both OCI‐Ly3 and OCI‐Ly1 cell lines, as evidenced by enhanced proliferation and reduced apoptosis, while the FOXK1‐R191K mutant restored DOX sensitivity (Figure 7A–C). Similarly, FOXK2‐WT overexpression promoted DOX resistance, whereas the FOXK2‐R144K mutant, when combined with DOX treatment, significantly inhibited proliferation and increased apoptosis in these cell lines (Figure 7D–F). These findings demonstrate that methylation of either FOXK1 or FOXK2 promotes DOX resistance in DLBCL cells, and that blocking these methylation events through FOXK1‐R191K/FOXK2‐R144K mutations can resensitise the cells to DOX treatment.

We next sought to determine whether PRMT7‐mediated chemoresistance functionally depends on FOXK1/FOXK2 and the downstream effector DVL2. Depletion of FOXK1/FOXK2 sensitised DOX‐resistant DLBCL cells to DOX treatment. Importantly, in the FOXK1/FOXK2‐deficient background, PRMT7 overexpression no longer enhanced cellular tolerance to DOX, suggesting that FOXK1/FOXK2 serve as important downstream effector molecules through which PRMT7 exerts its pro‐resistance function (Figure S17A,B). Consistently, knockout of DVL2 also sensitised DLBCL‐resistant cells to DOX. Moreover, in the DVL2‐deficient background, PRMT7 overexpression failed to enhance cellular tolerance to DOX, indicating that DVL2 is likewise a critical downstream node in PRMT7‐mediated resistance (Figure S17C,D). Collectively, these results establish that PRMT7‐driven chemoresistance is mechanistically dependent on the FOXK1/FOXK2–DVL2 signalling axis.

### 3.8 Blocking methylation at FOXK1 and FOXK2 restores DOX sensitivity and suppresses DLBCL progression

**FIGURE 8:**
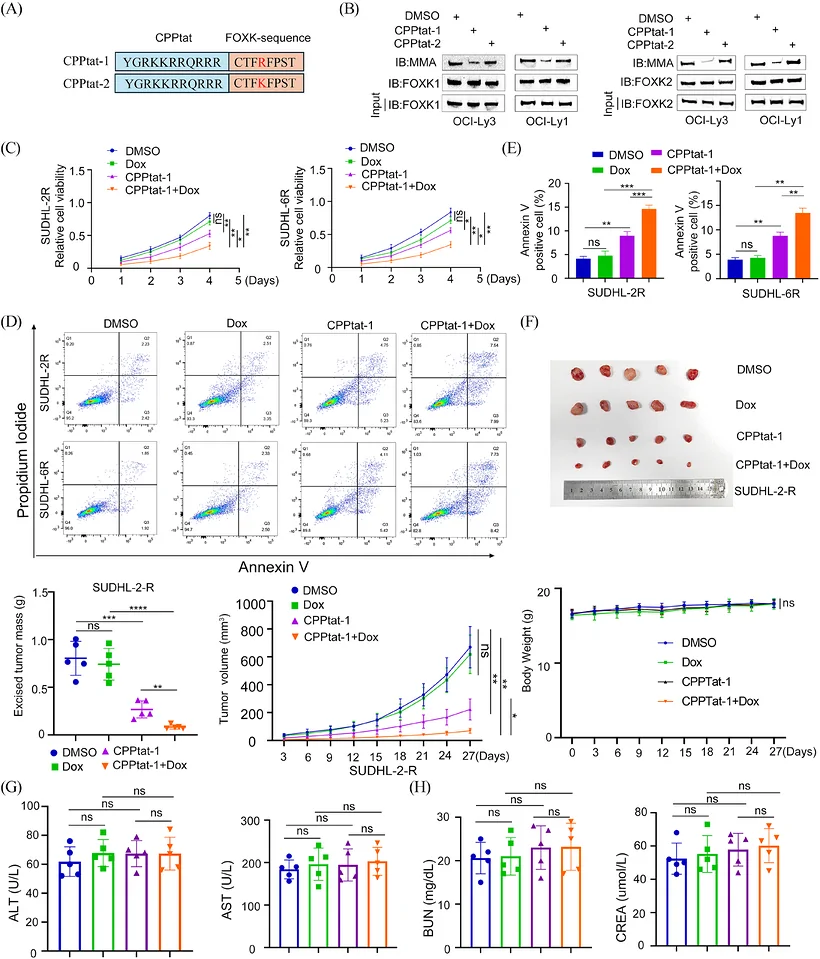
Blocking methylation at FOXK1‐R191 and FOXK2‐R144 restores doxorubicin sensitivity and suppresses DLBCL progression. (A) A cell‐penetrating peptide was fused with the conserved sequence shared by FOXK1 and FOXK2 (CTFRFPST) to generate the wild‐type (CPPtat‐1) or the R‐methylation site mutant (CPPtat‐2). (B) OCI‐ly3 and OCI‐ly1 cells were treated with CPPtat‐1 or CPPtat‐2, and IP was performed to detect the MMA levels of FOXK1 and FOXK2. The data are representative of three independent experiments. (C) SUDHL‐2R and SUDHL‐6R cells were treated with CPPtat‐1 and doxorubicin, and cell proliferation was assessed using a CCK8 assay. The experiments were repeated three independent times. Two‐way ANOVA followed by Bonferroni's post hoc test. (D and E) SUDHL‐2R and SUDHL‐6R cells were treated with CPPtat‐1 and doxorubicin, and apoptosis was detected using the Annexin V‐FITC/PI assay. The experiments were repeated three independent times. One‐way ANOVA followed by Tukey's multiple comparison test (E). The data are representative of three independent experiments (D). (F) Nude mice received subcutaneous injections of SUDHL‐2R cells into the left flank. The mice were treated with or without CPPtat‐1 and doxorubicin. Tumour volume and body weight of mice were measured every 3 days. On Day 27, tumours were harvested, photographed and weighed. *n* = 5 mice; data are representative of three independent experiments. Statistical analyses were performed using two‐way ANOVA followed by Bonferroni's post hoc test for tumour volume curves and body weight, and one‐way ANOVA followed by Tukey's multiple comparison test for excised tumour weights. (G and H) Serum biochemical markers of liver and kidney function were assessed on Day 27. (G) Liver function indicators, including ALT and AST. (H) Kidney function indicators, including BUN and CREA. Data are representative of three independent experiments. One‐way ANOVA followed by Tukey's multiple comparison test.

Given that PRMT7‐mediated methylation of FOXK1 and FOXK2 contributes to DOX resistance in DLBCL cells, we next investigated whether pharmacological interference with FOXK1/FOXK2 methylation could restore DOX sensitivity. Notably, FOXK1 and FOXK2 share an identical sequence motif (CTFKFPRT) surrounding their PRMT7‐targeted arginine residues. Based on this sequence similarity, we designed a competitive peptide‐based strategy to simultaneously inhibit FOXK1 and FOXK2 methylation. The FOXK‐derived peptide containing the CTFKFPRT sequence was fused to the TAT cell‐penetrating peptide (CPPTAT)^24^, ^25^ to generate CPPtat‐1. As a control, the corresponding peptide in which the target arginine residue was replaced by serine (CTFKFPST) was similarly fused to CPP‐TAT to generate CPPtat‐2 (Figure 8A).

We first evaluated the ability of these peptides to interfere with FOXK1/FOXK2 methylation. CPPtat‐1 markedly reduced the methylation of both FOXK1 and FOXK2, whereas the control peptide CPPtat‐2 had no detectable effect (Figure 8B). Consistent with the functional role of FOXK1/FOXK2 methylation, Co‐IP assays showed that CPPtat‐1 impaired the interaction of FOXK1/FOXK2 with DVL2 and subsequently suppressed Wnt/β‐catenin signalling activity (Figure S18A–D). We next assessed whether CPPtat‐1 could enhance the response of DOX‐resistant DLBCL cells to DOX. SUDHL‐2R and SUDHL‐6R cells were treated with CPPtat‐1, DOX or their combination. CCK‐8 and apoptosis assays demonstrated that the combination of CPPtat‐1 and DOX produced a greater inhibitory effect on cell viability and induced more pronounced apoptosis than either treatment alone (Figure 8C–E), indicating that inhibition of FOXK1/FOXK2 methylation enhances the sensitivity of resistant DLBCL cells to DOX.

We further evaluated the therapeutic efficacy of this combination in vivo. CPPtat‐1 significantly potentiated the tumour‐suppressive effect of DOX in DLBCL xenograft models (Figures 8F and S19A). Importantly, no significant differences in body weight were observed among the treatment groups throughout the experimental period. Moreover, serum biochemical analyses revealed no significant alterations in liver or renal function parameters among the groups (Figures 8G and S19B,C), suggesting that the combination treatment did not cause overt systemic toxicity under the experimental conditions used. Taken together, CPPtat‐1 serves as a proof‐of‐concept agent that enhances DLBCL chemosensitivity to DOX through inhibition of FOXK1/FOXK2 methylation, underscoring its favourable prospects for clinical translation.

## 4 DISCUSSION

This study reveals the critical role of the PRMT7–FOXK1/FOXK2–DVL2–Wnt/β‐catenin axis in DOX resistance in DLBCL. Our results demonstrated that PRMT7‐mediated methylation of FOXK1 (R191) and FOXK2 (R144) not only enhances their binding affinity with DVL2 but also promotes DVL2 nuclear translocation, thereby aberrantly activating the Wnt/β‐catenin signalling pathway. Notably, a bioactive peptide targeting FOXK1/FOXK2 methylation effectively inhibits this process and potentiates the anti‐DLBCL efficacy of DOX.

PRMT7 is a type III PRMT that is aberrantly expressed in multiple cancer types and has been implicated in diverse aspects of tumour progression.^8^, ^26^, ^27^ In the present study, we found that PRMT7 was highly expressed in DLBCL cells and promoted DLBCL growth both in vitro and in vivo, whereas PRMT7 depletion markedly impaired tumour growth. DOX is a key component of standard chemotherapy regimens for DLBCL; however, the development of drug resistance remains a major obstacle to durable therapeutic responses.^28^, ^29^ Our findings identify PRMT7 as an important regulator of DOX resistance in DLBCL, as increased PRMT7 expression reduced cellular sensitivity to DOX, whereas PRMT7 depletion enhanced the response to DOX. These observations extend the oncogenic role of PRMT7 beyond its effects on tumour growth and suggest that aberrant PRMT7 activity may contribute to therapeutic resistance in DLBCL.

PRMT7 can regulate cellular processes through the methylation of both histone and non‐histone substrates.^8^ Here, we identified FOXK1 and FOXK2 as previously unrecognised non‐histone substrates of PRMT7 and mapped the PRMT7‐dependent methylation sites to R191 of FOXK1 and R144 of FOXK2. Interestingly, the sequences surrounding these methylated arginine residues are highly similar, suggesting that PRMT7 may recognise a shared local sequence feature in FOXK1 and FOXK2. Despite the high structural similarity between FOXK1 and FOXK2, knockdown of one member did not cause significant compensatory expression changes in the other, nor did it affect their respective binding to DVL2 (Figure S20A,B). Importantly, our findings reveal a previously unrecognised functional consequence of FOXK1/FOXK2 methylation. Methylation of FOXK1 at R191 and FOXK2 at R144 enhanced their interactions with DVL2, whereas methylation‐deficient mutations markedly impaired these interactions. Given the established role of FOXK1/FOXK2 in facilitating DVL2 nuclear translocation,^22^ our results indicate that PRMT7‐dependent methylation provides an additional post‐translational layer of regulation that controls the FOXK1/FOXK2–DVL2 interaction and subsequent DVL2 nuclear localisation.

Notably, PRMT7 did not substantially alter FOXK1/FOXK2 expression, and methylation‐deficient mutations did not affect the subcellular localisation of FOXK1/FOXK2 themselves. Thus, rather than regulating FOXK1/FOXK2 abundance or localisation, PRMT7‐mediated methylation appears to modulate their functional interaction with DVL2. Functionally, methylation‐deficient FOXK1/FOXK2 mutants failed to confer the same degree of DOX resistance as their wild‐type counterparts, supporting the importance of these methylation events in the chemoresistant phenotype.

DVL2 is a central component of Wnt signalling, and its nuclear localisation has been implicated in the transcriptional regulation of Wnt/β‐catenin target genes.^23^, ^30^ Nuclear DVL can participate in the β‐catenin/TCF transcriptional machinery and facilitate Wnt target gene transcription.^23^ Our study confirms that arginine methylation of FOXK1/FOXK2 enhances their binding ability to DVL2 and promotes the entry of DVL2 into the nucleus, thereby activating the Wnt/β‐catenin pathway.

Several limitations of this study should be acknowledged. First, our findings on DOX response remain to be validated in clinical specimens, and the current conclusions are therefore restricted to cellular and animal models. Further investigation is warranted to address this limitation. Second, we currently lack clinical data examining the correlation between PRMT7 expression and refractory/relapsed disease in DLBCL patients. Consequently, the potential role of PRMT7 in mediating treatment resistance and its prognostic implications for this high‐risk subgroup remain unexplored, and future studies with dedicated longitudinal cohorts are required to address this gap. Third, most mechanistic experiments were conducted using established DLBCL cell lines, which may not fully capture the molecular and biological heterogeneity of human DLBCL. Validation in patient‐derived systems, including primary DLBCL cells, would strengthen the clinical relevance of the proposed signalling axis. Fourth, our in vivo studies primarily relied on subcutaneous xenograft models. Although these models enabled evaluation of tumour growth and therapeutic response, they do not fully recapitulate the anatomical, stromal and immune microenvironment of human DLBCL. Future studies using more clinically relevant models, including patient‐derived xenografts and appropriate immunocompetent models, will therefore be important. Finally, although the CPPtat‐1‐based strategy demonstrated proof‐of‐concept therapeutic activity, its development remains at an early stage. Further optimisation of peptide stability, pharmacokinetic properties and target specificity, together with comprehensive safety and efficacy assessments, will be necessary before its translational potential can be fully evaluated.

In conclusion, our study identifies a previously unrecognised mechanism of DOX resistance in DLBCL in which PRMT7 methylates FOXK1 at R191 and FOXK2 at R144, thereby strengthening their interaction with DVL2 and promoting DVL2 nuclear translocation. Nuclear DVL2 subsequently enhances Wnt/β‐catenin transcriptional activity, ultimately contributing to the chemoresistant phenotype. Furthermore, competitive interference with FOXK1/FOXK2 methylation disrupted this signalling cascade and enhanced the antitumour efficacy of DOX in preclinical models.

*Conclusion*: Our study not only reveals the mechanistic involvement of the PRMT7–FOXK1/FOXK2–DVL2–Wnt/β‑catenin axis in DLBCL DOX resistance, but also develops a methylation‐blocking peptide targeting FOXK1/FOXK2, which acts synergistically with DOX to augment its antitumour efficacy. As a proof‐of‐concept agent, this peptide offers preliminary promise for clinical use, though its safety and efficacy await further confirmation.

## AUTHOR CONTRIBUTIONS

X. Y. Z., J. S. Z., Y. T., Y. Q. W. and W. Y. carried out the experiments and analysed the data. X. Y. Z., J. S. Z. and Y. T. wrote and the manuscript. Y. S., Z. W. and A. G. L. conceived and supervised the project, wrote and revised the manuscript. All authors read and approved of the final manuscript.

## CONFLICT OF INTEREST STATEMENT

The authors declare no conflicts of interest.

## CONSENT FOR PUBLICATION

All authors agreed on the manuscript for publication.

## ETHICS STATEMENT

Human specimen collection was conducted in accordance with the principle of informed consent and complied with the ethical requirements of the Declaration of Helsinki. Ethical approval for this study was obtained from the Ethics Committee of Union Hospital, Huazhong University of Science and Technology. All animal experimental procedures were approved by the Animal Ethics Committee of Tongji Medical College, Huazhong University of Science and Technology and strictly complied with laboratory animal welfare ethics guidelines.

## Supporting information



Supporting Information: ctm270802‐sup‐0001‐SuppMat

## Data Availability

All data generated or analysed during this study are included in this article and its Supplementary Information Files.
