# Supplementary material for: PRMT7‐mediated arginine methylation of FOXK activates Wnt/β‐catenin signalling to drive doxorubicin resistance in diffuse large B‐cell lymphoma
Source: Clin Transl Med. 2026 Sep 2;16(9):e70802. doi: 10.1002/ctm2.70802 (PMC13535836; doi:10.1002/ctm2.70802)
Supplement: Supplementary file 1 [file CTM2-16-e70802-s001.docx]

**PRMT7-Mediated Arginine Methylation of FOXK Activates Wnt/β-catenin Signaling to Drive Doxorubicin Resistance in Diffuse Large B-Cell Lymphoma**

**Figure S1. Stable Maintenance of Doxorubicin Resistance upon Drug Removal**

(A-B) IC50 of doxorubicin in SUDHL-2/SUDHL-2R and SUDHL-6/SUDHL-6R cells were determined by CCK-8 assay. The experiments were repeated three independent times. Student’s test.

(C-D) The IC50 values of doxorubicin (nM) were measured in SUDHL-2 and SUDHL-6 parent cells (P), doxorubicin-resistant cells (R), and doxorubicin-withdrawn cells (R dox withdrawal) at 2 weeks (2W) and 5 weeks (5W) post-treatment using CCK-8 assays. The experiments were repeated three independent times. One-way ANOVA followed by Tukey's multiple comparison test.

**Figure S2 PRMT7 is highly expressed in DLBCL cell lines.**

(A) Relative mRNA expression levels of PRMT7 were determined by RT-qPCR in normal control samples (N1, N2, N3) and various DLBCL cell lines. The experiments were repeated three independent times. Statistical significance was determined using one-way ANOVA followed by Tukey's multiple comparison test.
(B) Western blotting analysis of PRMT7 protein expression in the above groups. Data are representative of three independent experiments.

**Figure S3 Overexpression of PRMT7 promotes DLBCL cell proliferation.**

(A) PRMT7 expression levels detected by Western blotting after overexpression of Flag-PRMT7 in SUDHL-2 and SUDHL-6 cells. Data are representative of three independent experiments.
(B) Cell proliferation detected by CCK8 assay after overexpression of Flag-PRMT7 in SUDHL-2 and SUDHL-6 cells. The experiments were repeated three independent times. Two-way ANOVA followed by Bonferroni’s post hoc test.

(C) Apoptosis rate detected by flow cytometry after overexpression of Flag-PRMT7 in SUDHL-2 and SUDHL-6 cells. The experiments were repeated three independent times. Student's t test.

(D) SUDHL-2 and SUDHL-6 cells overexpressing PRMT7 were injected into the left flank of nude mice. After 18 days, tumor tissues were collected for: photographic documentation, weight measurement (n=5 mice). Data are representative of three independent experiments. Statistical analyses were performed using two-way ANOVA followed by Bonferroni’s post hoc test for tumor volume curves and body weight, and one-way ANOVA followed by Tukey's multiple comparison test for excised tumor weights.

**Figure S4. PRMT7 modulates doxorubicin sensitivity in DLBCL**

(A) IC50 of doxorubicin was determined by CCK-8 assay in SUDHL-2 and SUDHL-6 cells after PRMT7 overexpression. The experiments were repeated three independent times. Student's t test.
(B) IC50 of doxorubicin was determined by the CCK-8 assay in OCI-Ly3 and OCI-Ly1 cells after PRMT7 knockout. The experiments were repeated three independent times. One-way ANOVA followed by Tukey's multiple comparison test.

**Figure S5. PRMT7 knockout enhances the proliferation-inhibitory effect of doxorubicin on DLBCL cells.**

(A) PRMT7-knockout OCI-ly3 and OCI-Ly1 cells were treated with doxorubicin, and cell proliferation was assessed using a CCK8 assay. The experiments were repeated three independent times. Two-way ANOVA followed by Bonferroni’s post hoc test.

(B-C) PRMT7-knockout OCI-ly3 and OCI-Ly1 cells were treated with doxorubicin, and the proportion of apoptotic cells was detected using Annexin V-FITC/PI assay. The experiments were repeated three independent times. One-way ANOVA followed by Tukey's multiple comparison test.

**Figure S6**

(A) Nuclear-cytoplasmic fractionation assays verifying the nuclear entry ability of FOXK1 and FOXK2. Cells were transfected with indicated plasmids. Cytoplasmic and nuclear fractions were subjected to Western blotting. The del-NLS mutants were predominantly localized in the cytoplasm and failed to enter the nucleus. Data are representative of three independent experiments.
(B) Cells were transfected with the indicated plasmids, and the interaction of FOXK1 with PRMT7 and DVL2 was examined by Co-IP. Data are representative of three independent experiments.

**Figure S7. Mutation of R191 or R144 eliminates methylation of FOXK1 or FOXK2, respectively.**

(A) Group-based Prediction System-Methylation Site Predictor was used to predict arginine methylation sites on FOXK1.

(B) Group-based Prediction System-Methylation Site Predictor was used to predict arginine methylation sites on FOXK2.

(C) Conservation analysis of R191 residue of FOXK1 and R144 residue of FOXK2.

(D) HA-FOXK1-WT and HA-FOXK1-R191K plasmids were transfected into SUDHL-2 and SUDHL-6 cells. Cells were treated with SGC3027, and IP was performed to detect MMA levels of FOXK1. Data are representative of three independent experiments.

(E) HA-FOXK2-WT and HA-FOXK2-R144K plasmids were transfected into SUDHL-2 and SUDHL-6 cells. Cells were treated with SGC3027, and IP was performed to detect MMA levels of FOXK2. Data are representative of three independent experiments.

**Figure S8. FOXK1 and FOXK2 undergo arginine monomethylation.**

SUDHL-2 and SUDHL-6 cells were transfected with indicated plasmids. IP was performed to detect MMA, symmetrically dimethylated (SDMA), and asymmetrically dimethylated (ADMA) arginine levels on FOXK1/FOXK2. Data are representative of three independent experiments.

**Figure S9. PRMT7 has no effect on the expression levels of FOXK1 or FOXK2.**

(A-B) qRT-PCR analysis of FOXK1 and FOXK2 mRNA expression levels following PRMT7 knockout. The experiments were repeated three independent times. Student’s t-test.

(C) Western blotting analysis of FOXK1 or FOXK2 protein expression levels after PRMT7 knockout. Data are representative of three independent experiments.

(D-E) qRT-PCR was used to assess the mRNA levels of FOXK1 or FOXK2 following PRMT7 overexpression. The experiments were repeated three independent times. Student’s t-test.

(F) Western blotting analysis of FOXK1 or FOXK2 protein levels after PRMT7 overexpression. Data are representative of three independent experiments.

(G-H) The mRNA levels of FOXK1 and FOXK2 were determined by qRT-PCR after transfection with the indicated plasmids. The experiments were repeated three independent times. One-way ANOVA followed by Tukey's multiple comparison test.

(I) The protein levels of FOXK1 and FOXK2 were evaluated by Western blotting in cells expressing the indicated plasmids. Data are representative of three independent experiments.

**Figure S10. Nuclear translocation of DVL2 depends on PRMT7 enzymatic activity**

(A) SUDHL-2 and SUDHL-6 cells were transfected with either Flag-PRMT7-WT or the catalytically inactive mutant Flag-PRMT7-LDIG. Co-IP was performed to assess the interaction between FOXK1/FOXK2 and DVL2. Data are representative of three independent experiments.

(B-C) SUDHL-2 and SUDHL-6 cells overexpressing Flag-PRMT7-WT or Flag-PRMT7-LDIG were subjected to nuclear and cytoplasmic fractionation to detect the localization of DVL2 (B). The indicated cells were transferred to slides for immunofluorescence. Representative images and quantification of 20 cells are shown in the figure (C). Scale bar: 10 μm. The experiments were repeated three independent times. One-way ANOVA followed by Tukey's multiple comparison test.

**Figure S11. Knockout of PRMT7 does not affect the mRNA and protein expression levels of DVL2.**

(A) The mRNA levels of DVL2 were assessed by qRT-PCR following PRMT7 knockout in SUDHL-2 and SUDHL-6 cells. The experiments were repeated three independent times. One-way ANOVA followed by Tukey's multiple comparison test. (B) The protein expression levels of DVL2 were examined by Western blotting after PRMT7 knockout. Data are representative of three independent experiments.

**Figure S12. Methylation of FOXK1 and FOXK2 does not affect their subcellular localization.**

Cells were transfected with indicated plasmids. Cytoplasmic and nuclear fractions were prepared and subjected to Western blotting. Data are representative of three independent experiments.

**Figure S13. Wnt/β-catenin pathway activation requires PRMT7 enzymatic activity**

(A-B) SUDHL-2 and SUDHL-6 cells transfected with Flag-PRMT7-WT or the catalytic mutant Flag-PRMT7-LDIG were analyzed by: qRT-PCR for Axin2, c-Myc, and Cyclin D1 mRNA levels (A), the experiments were repeated three independent times. One-way ANOVA followed by Tukey's multiple comparison test. Western blotting for corresponding protein expression (B), data are representative of three independent experiments.

(C) β-catenin transcriptional activity was measured by a luciferase reporter assay in SUDHL-2/SUDHL-6 cells expressing PRMT7-WT or LDIG mutant. The experiments were repeated three independent times. One-way ANOVA followed by Tukey's multiple comparison test.

**Figure S14. FOXK1/FOXK2 methylation does not significantly affect the expression of β-catenin.**

(A-B) SUDHL-2 and SUDHL-6 cells were transfected with indicated plasmids. The expressions of β-catenin and phosphorylation-β-catenin were detected by western blotting. Data are representative of three independent experiments.

(C) The protein expression levels of β-catenin and phosphorylated β-catenin (p-β-catenin) were examined by Western blotting following PRMT7 knockout. Data are representative of three independent experiments.

(D) The protein expression levels of β-catenin and p-β-catenin were assessed by Western blotting after transfection with the indicated plasmids. Data are representative of three independent experiments.

**Figure S15. Overexpression of PRMT7 enhances doxorubicin resistance in a Wnt signaling-dependent manner.**

(A) Dose-response curves and IC50 values of doxorubicin in SUDHL-2R and SUDHL-6R cells under the indicated conditions. The experiments were repeated three independent times. One-way ANOVA followed by Tukey's multiple comparison test.
(B) Cell proliferation curves of SUDHL-2R and SUDHL-6R cells were determined by CCK-8 assays under the four indicated conditions. The experiments were repeated three independent times. Two-way ANOVA followed by Bonferroni’s post hoc test.

**Figure S16. FOXK1/FOXK2 methylation promotes doxorubicin resistance in DLBCL cells**

(A-B) OCI‑LY3 and OCI‑LY1 cells were transfected with either HA-FOXK1-WT or methylation-deficient mutant HA-FOXK1-R191K and were treated with doxorubicin. Dose‑response curves and IC₅₀ values of doxorubicin in OCI‑LY3 and OCI‑LY1 cells were determined. The experiments were repeated three independent times. One-way ANOVA followed by Tukey's multiple comparison test (B).

(C-D) OCI‑LY3 and OCI‑LY1 cells were transfected with either HA-FOXK2-WT or methylation-deficient mutant HA-FOXK1-R144K and were treated with doxorubicin. Dose‑response curves and IC₅₀ values of doxorubicin in OCI‑LY3 and OCI‑LY1 cells were determined. The experiments were repeated three independent times. One-way ANOVA followed by Tukey's multiple comparison test (D).

**Figure S17. PRMT7 overexpression promotes drug resistance in DLBCL cells in a FOXK- and DVL2-dependent manner.**

(A and C) Dose-response curves and IC50 values of doxorubicin in SUDHL-2R and SUDHL-6R cells. Left panels show relative cell viability curves upon treatment with various concentrations of Dox under the indicated conditions. The experiments were repeated three independent times. One-way ANOVA followed by Tukey's multiple comparison test.

(B and D) Cell proliferation curves of SUDHL-2R and SUDHL-6R cells determined by CCK-8 assays under the four indicated conditions. The experiments were repeated three independent times. Two-way ANOVA followed by Bonferroni’s post hoc test.

**Figure S18. CPPtat-1 inhibits the Wnt/β-catenin signaling pathway**

(A) OCI-Ly3 and OCI-Ly1 cells treated with either CPPtat-1 and CPPtat-2 were subjected to Co-IP to assess interactions between DVL2 and FOXK1/FOXK2. Data are representative of three independent experiments.

(B-C) Wnt/β-catenin downstream targets were evaluated in treated cells by: qRT-PCR for Axin2, c-Myc, and Cyclin D1 mRNA levels (B), the experiments were repeated three independent times. One-way ANOVA followed by Tukey's multiple comparison test (B); Western blotting for corresponding protein expression (C), data are representative of three independent experiments.

(D) β-catenin transcriptional activity was measured by a luciferase reporter assay in CPPtat-1- or CPPtat-2-treated cells; the experiments were repeated three independent times. One-way ANOVA followed by Tukey's multiple comparison test.

**Figure S19. Blocking methylation at FOXK1-R191 and FOXK2-R144 restores**

**doxorubicin sensitivity and suppresses DLBCL progression.**

(A) Nude mice received subcutaneous injections of SUDHL-6R cells into the left flank. The mice were treated with or without CPPtat-1 and doxorubicin. Tumor volume and body weight of mice were measured every 3 days. On day 27, tumors were harvested, photographed, and weighed. n=5 mice; data are representative of three independent experiments. Statistical analyses were performed using two-way ANOVA followed by Tukey's multiple comparison test for tumor volume curves and body weight, and one-way ANOVA followed by Tukey's multiple comparison test for excised tumor weights.

(B-C) Serum biochemical markers of liver and kidney function were assessed on day 27. (B) Liver function indicators, including ALT and AST. (C) Kidney function indicators, including BUN and CREA. Data are representative of three independent experiments. One-way ANOVA followed by Tukey's multiple comparison test.

**Figure S20. FOXK1 and FOXK2 did not exhibit significant compensatory effects.**

(A) The expression levels of FOXK2 were examined by Western blotting following FOXK1 knockdown (left panel). The expression levels of FOXK1 were examined by Western blotting following FOXK2 knockdown (right panel). Data are representative of three independent experiments.

(B) Co-IP assays were performed to evaluate the binding of FOXK2 to DVL2 upon FOXK1 knockdown, and the binding of FOXK1 to DVL2 upon FOXK2 knockdown, respectively. Data are representative of three independent experiments.

**Table 1. The characteristics of DLBCL patients.**

|  | **PRMT7 expression** | |  |
| --- | --- | --- | --- |
|  | Low (n=37) | High (n=26) | P-value |
| **Age (years)** |  |  | 0.889 |
| >60 years | 12 | 8 |  |
| ≤60 years | 25 | 18 |  |
| **Gender** |  |  | 0.620 |
| Male | 19 | 15 |  |
| Female | 18 | 11 |  |
| **IPI score** |  |  | 0.555 |
| 0–2 | 20 | 16 |  |
| 3–5 | 17 | 10 |  |
| **Disease stage** |  |  | 0.966 |
| I–II | 13 | 9 |  |
| III–IV | 24 | 17 |  |
| **ECOG PS** |  |  | 0.558 |
| ≤1 | 26 | 20 |  |
| >1 | 11 | 6 |  |
| **Extranodal site(s)** |  |  | 0.264 |
| 1 or 0 | 22 | 19 |  |
| >1 | 15 | 7 |  |
| **LDH** |  |  | 0.536 |
| ≤ULN (270 µmol/L) | 17 | 14 |  |
| >ULN (270 µmol/L) | 20 | 12 |  |
| **Hans classification** |  |  | 0.941 |
| GCB | 16 | 11 |  |
| Non‐GCB | 21 | 15 |  |

**Table 2. The primer sequence of genes**

| **Gene** | **Forward primer** | **Reverse primer** |
| --- | --- | --- |
| **FOXK1** | 5ʹ-CATTACCCCTACTACCGGACG-3ʹ | 5ʹ-GTAACGGTTCAAAGAGAGGTTGT-3ʹ |
| **FOXK2** | 5ʹ-GGACTAAACGGCACCAGAAA-3ʹ | 5ʹ-CCATCCAAGCCTCCATCATA-3ʹ |
| **AXIN2** | 5ʹ-GTCCAGCAAAACTCTGAGGG-3ʹ | 5ʹ-CTGGTGCAAAGACATAGCCA-3ʹ |
| **CCND1** | 5ʹ- GACCTTCGTTGCCCTCTGT-3ʹ | 5ʹ- TGAGGCGGTAGTAGGACAGG-3ʹ |
| **MYC** | 5'-GCCTCAGAGTGCATCGAC-3' | 5'-TCCACAGAAACAACATCG-3' |
| **β-tubulin** | 5'-ACCAACCTACGGGGATCTGAA-3' | 5'-TTGACTGCCAACTTGCGGA-3' |
| **DVL2** | 5'-GAGGAAGAGACTCCCTACCTG-3' | 5'-CGGGCGTTGTCATCTGAAAT-3 |
